# CT brush and CancerZap!: two video games for computed tomography dose minimization

**DOI:** 10.1186/s12976-015-0003-4

**Published:** 2015-05-12

**Authors:** Graham Alvare, Richard Gordon

**Affiliations:** BioInformation Technology Laboratory, Department of Plant Science, University of Manitoba, E2-532 EITC, Winnipeg, R3T 2N2 MB Canada; Current address: Faculty of Medicine, University of Manitoba, Box 107, Winnipeg, Canada; Embryogenesis Center, Gulf Specimen Aquarium and Marine Laboratory, 222Clark Drive, Panacea, FL 32346 USA; C.S. Mott Center for Human Growth and Development, Department of Obstetrics and Gynecology, Wayne State University, 275 E. Hancock, Detroit, MI 48201 USA; Stellarray, 9210 Cameron Road Suite #300, Austin, TX 78754 USA

**Keywords:** Object dependent image processing, Computed tomography, Video games, X-ray dose reduction, Progressive compressive sensing, Crowdsourcing, Human computing

## Abstract

**Background:**

X-ray dose from computed tomography (CT) scanners has become a significant public health concern. All CT scanners spray x-ray photons across a patient, including those using compressive sensing algorithms. New technologies make it possible to aim x-ray beams where they are most needed to form a diagnostic or screening image. We have designed a computer game, CT Brush, that takes advantage of this new flexibility. It uses a standard MART algorithm (Multiplicative Algebraic Reconstruction Technique), but with a user defined dynamically selected subset of the rays. The image appears as the player moves the CT brush over an initially blank scene, with dose accumulating with every “mouse down” move. The goal is to find the “tumor” with as few moves (least dose) as possible.

**Results:**

We have successfully implemented CT Brush in Java and made it available publicly, requesting crowdsourced feedback on improving the open source code. With this experience, we also outline a “shoot ‘em up game” CancerZap! for photon limited CT.

**Conclusions:**

We anticipate that human computing games like these, analyzed by methods similar to those used to understand eye tracking, will lead to new object dependent CT algorithms that will require significantly less dose than object independent nonlinear and compressive sensing algorithms that depend on sprayed photons. Preliminary results suggest substantial dose reduction is achievable.

**Electronic supplementary material:**

The online version of this article (doi:10.1186/s12976-015-0003-4) contains supplementary material, which is available to authorized users.

## Background

We would like to introduce two computer games for x-ray computed tomography (CT), with the goal of capturing and using human intuition for reducing CT dose. The first game is based on a generalization of a common computer drawing tool, called the “brush”, which resembles art and drafting tools [1-6]. Here we create what we call a “CT brush”. We have implemented the core of the CT Brush game and made it challenging with increasing levels of difficulty. The second game, CancerZap! [7], designed based on watching absorbed grandchildren, allows players to shoot bursts of x-ray photons at “bad guys” (tumors) while trying to miss the “good guys” (normal tissues) as much as possible, with the caveat that initially you can’t see either, and so the user must start by “shooting in the dark”. As the images improve, you have to decide who is good or bad. Such games are an amalgam of the *Where’s Waldo* books [8,9] and the Battleship game [10-14]. Thus, CT Brush is a “puzzle game” and CancerZap! is a “first person shooter” game [15]. A form of the latter game, CancerZap!, has already found its way into XLCT (X-ray Luminescence CT) software for sparse molecular images [16] based on our previous suggestions [17]. A more general CancerZap! game is outlined here, but has not yet been implemented.

In both games, as in golf, “the goal is to play as few strokes per round as possible” [18]; or, in other words, the purpose of the game is to find the tumors with as low an x-ray dose (the game score) as possible. Both games mimic standard absorption/scattering x-ray imaging, but should be modifiable to create games that use x-ray phase contrast imaging, which promises substantial x-ray dose reduction in itself [19-21].

Present CT scanners use shotgun approaches, spraying the patient with x-rays and constructing an image from the projection data obtained in the process. The mathematics is generally linear and nonadaptive, including that of compressed sensing [22-29], although modern algorithms incorporate some *a priori* information such as positivity [30-33], smoothness [34], piecewise continuity [35-38], streak suppression [39,40], working around opaque objects [41-43], modeling [44-46], thickness of the patient versus angle of view [34,47,48], etc. Here we consider what we have called intelligently steered x-ray beams [17], the idea being that human intelligence might lead to detection of tumors at lower dose than shotgun-based algorithms. If so, we might be able to automate what people do, by recording and analyzing their search strategies.

In medicine we calculate computed tomography images to detect problems inside patients. While these scanners work, they do so at high x-ray dose, and the controversy over this cumulative dose to the population, 49% of the per capita dose in the USA [49], is hindering wider use of CT. There have been many algorithmic approaches to dose reduction [50-53], but in our opinion much further dose reduction should be possible. This is especially the case for our long term goal, which is the detection of premetastasis breast and other tumors [17]. We found that detection of 2 to 4 mm diameter premetastasis breast tumors, followed by their destruction, should lead to a greater than 99% cure rate [54]. Others subsequently estimated this target at 2.7 mm [55]. Our focus here is, then, on the detection of small tumors, rather than the quality of the general CT diagnostic image. The reduced resolution of iterative CT algorithms as dose is reduced [56] could be offset if the x-rays were directed more to the tumors being sought, as we propose here.

### Implementation

#### A 2D version of the CT brush

Please see Additional files 1, 2, and 3, which contain a binary JAR, the source code, and the documentation, respectively.

The simplest brush tool is the eraser, which has a given size and shape, and sets all pixels it encounters to zero as it is moved via mouse, joystick or track pad by the player [57]. The drawing brush tool likewise fills in a swath of pixels with a given value, design or color. Other tools have more subtle effects, such as “healing” (removing scratches, etc.), creating gradients, blurring edges or making smoke patterns as the tool moves. These tools can be used to create a digital painting from scratch, guided by the mind of the artist.

**Figure 1:**
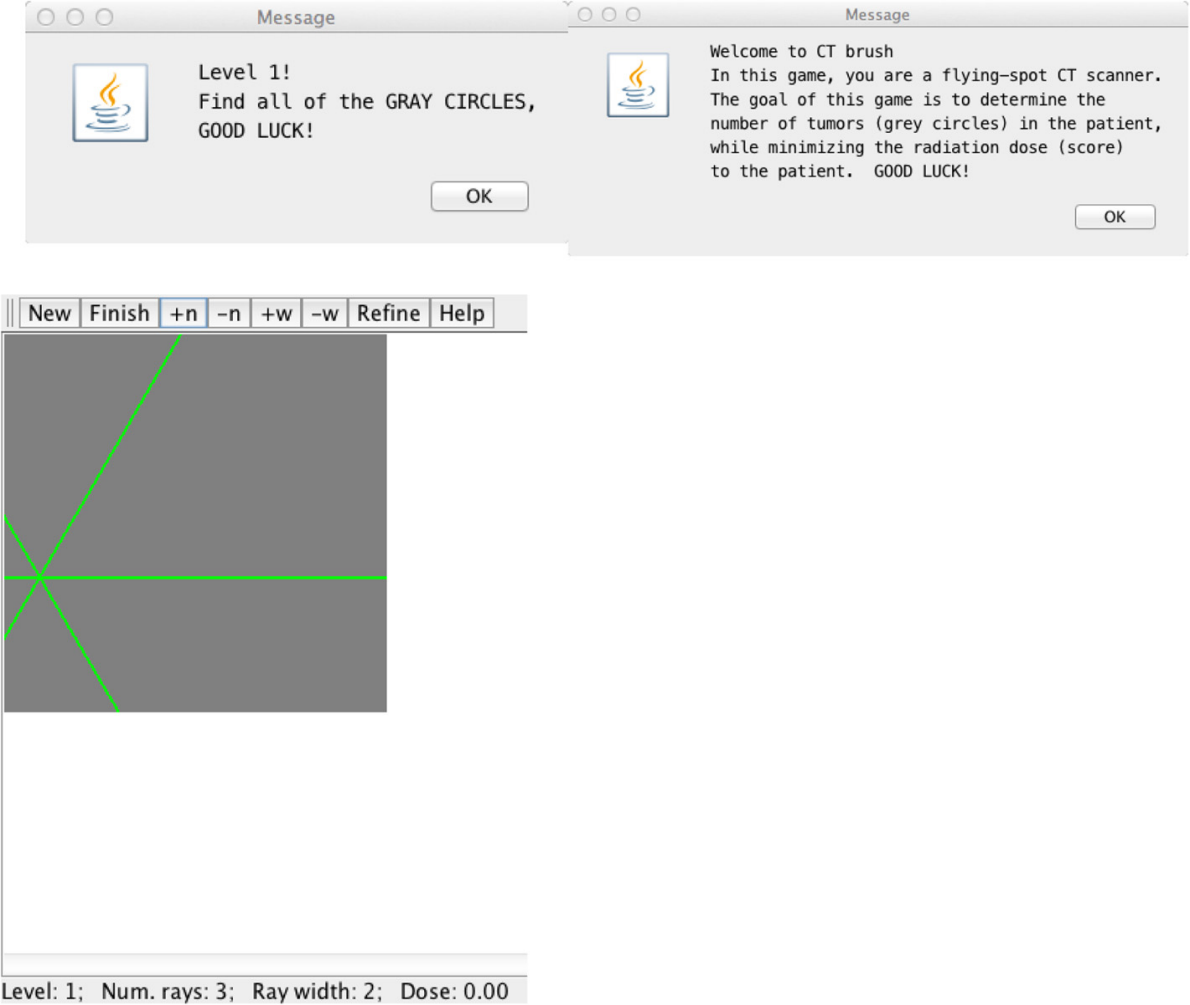
The welcoming messages and initially uniform image in the CT Brush game. Buttons are available on screen to the player for increasing or decreasing the number of rays (n) or the width of the rays (w) at any time during play. “New” starts a new game. “Refine” allows the user to run the iterative CT algorithm (MART) to convergence. This does not add any dose. The rays are initially all green, meaning no dose has yet been applied along them.

**Figure 2:**
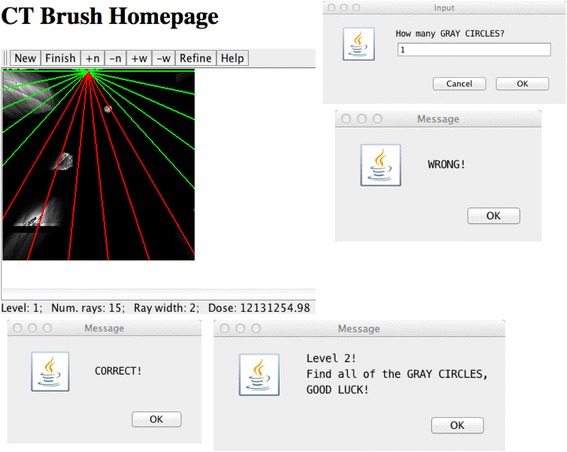
On hitting the “Finish” button, the player is asked to state how many gray circles have been found. Here play was terminated early, and a wrong guess was made. Note that some of the rays are red, meaning that they have been used before, and are therefore not adding to the dose. The dose used so far is reported continuously on the bottom, along with the current number of rays and ray width. A correct guess advances one to the next level of difficulty. Here the image size is 256 x 256 pixels.

**Figure 3:**
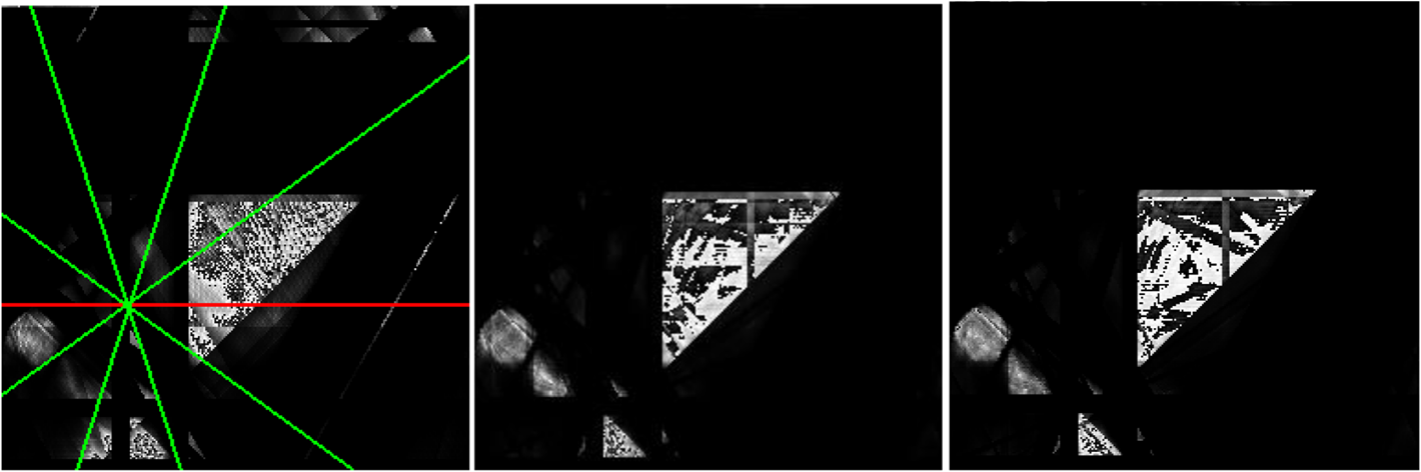
*Left*: This depicts the star-brush in the CT Brush game, as seen by a player, after playing a while on a 256x256 pixels canvas. Both real objects and artifacts appear in the image, but the player does not know which are which. The red lines represent selected rays (i.e., rays which have already been irradiated and analyzed), while the green lines represent available, unselected rays. These lines are shown dynamically as the player moves the brush. If the mouse button is down, the green rays become selected, turn red, and the dose is increased. Early in the game, one is playing “blind”. *Middle*: further use of the CT brush starts to bring out more of the structure of the hidden image, and (what will later prove to be) artifacts decrease. *Right*: refinement of the image by further play clearly brings out the gray circle, which represents a possible tumor. The player might decide to stop at this point and report the apparent count of tumors.

A CT brush consists of a “star” pattern of x-ray beams (rays) through a given point in the patient (Figures 1, 2 and 3). The point at which all beams in the brush intersect is called the “central point”. As the brush moves, both the central point and all of the star lines through it are processed. The simplest design is to have a set of fixed, intersecting beams and move them from point to point within the patient, perhaps turning them on and off as we go. This would allow us to control the dose. This could be implemented in hardware using modern x-ray arrays, perhaps using Wolter lenses [58]. Note that while we are confining CT Brush to 2D images, it could readily be generalized to 3D, with, in general, a sharper point spread function for the images [17,59]. However, it would best be driven by a 3D joystick [60] and the images displayed in 3D.

Dose is controlled by two factors: the number of rays involved in the star of the CT brush, and by the motion of the brush when the player holds the mouse button down. As the player moves the CT brush with the mouse button down, all points along the rays of the star-brush receive radiation, i.e., all of those rays are labeled as “selected”. However, to minimize redundant dose, we do not count selected rays twice. This is because the value of a given projection in this game is static, and so reprojecting a ray will not yield any increase in image quality for the player. The player can see this effect, because all of the projection lines in the star, which have not yet been irradiated, will be displayed as green; meanwhile, any projection lines in the star of the CT brush, which have already been irradiated, will be displayed as red. Green means “available for selection” and red means “already selected”. Thus the player is prevented from adding dose that could not improve the image. Again, this protection from extra dose when a ray is selected twice could be implemented in computer driven x-ray hardware.

If the player has a mouse with a wheel, the number of rays can be increased or decreased by turning it. Otherwise there are screen buttons for increasing/decreasing the number of rays. Screen buttons are also provided for increasing/decreasing the brush width. The dose per star increases in proportion to both its number of rays and its ray width. No detail is lost, since the rays that are bundled in a wide brush are calculated separately. In the current implementation, the views are equally spaced all the way around. Options could be added for limited angle range [30,61-72] and for rotating the CT brush, thereby generating a fresh set of rays, despite using the same number of views in the star.

Using the CT brush, the player can select which subset of all possible rays across the image to use for the MART algorithm. As the player brushes the hidden image, a “canvas image” is generated. This canvas image is constructed using all of the ray sum values from the rays selected thus far as the brush was moved. Therefore, the maximum dose occurs if the player brushes all points in the canvas, with 180 rays in the star (the maximum number of rays we have allowed in the computer program, each line representing one degree around the central point). In other words, an image produced this way would be equivalent to an image made using 180 projected, parallel views equally spaced by 1 degree, and the same CT algorithm. In summary, the user of CT Brush is selecting a subset of all possible rays. The smaller that subset, the lower the dose.

The CT brush, while having a focal point (the “central point”), extends across the whole image, because each ray enters and exits at edges of the image (Figure 1, 2, and 3). Therefore, as the brush is moved with the mouse down, data is acquired for every pixel in the hidden image that is intersected by the brush’s star pattern. The trajectory of the central point is tracked for later analysis. The raysum data is accumulated over time; so, as the CT brush is moved, all points in the canvas image (i.e. the patient), touched by any ray in the brush, may be updated to new values. These effects will fall off as the distance from the current location of the central point of the CT brush increases [73].

The CT algorithm we used, MART, is iterative and ray based. Because the program’s algorithms have to be run in real-time on consumer personal computers, some compromises were necessary, such as only doing refinement iterations when the player clicks the “refine” button. Refining does not increase dose. The pattern of use of the refine button is recorded using the tracking feature, permitting its later automation.

#### CT brush playing levels

To facilitate unlimited playability, the CT Brush game generates levels for the player to solve. Each “level” (i.e. level of difficulty) in the CT Brush game corresponds to a hidden image comprised of gray and whole-tone (either white or black, depending on the background color) objects. As the user progresses, he or she may access canvases that are larger in height or width (or both), by up to 128 pixels per 5 levels. These larger canvases will appear randomly. Because the maximum number of objects is calculated randomly based on the dimension of the canvas, larger hidden images will generally be more complex. In addition, beyond level 8, there is a 1/6 chance for the grayscale of a level being inverted. The number of shapes per level is calculated by: mindim/64 + random((mindim/11) - (mindim/64)) -- where mindim = the minimum dimension (height or width) in pixels. The reason higher levels are generally (but not always) harder is because the larger the image sizes available, the more likely the user is going to have a larger image. The randomness also provides the user with the occasional easy level.

The goal of each level in the game is to find the number of gray circles in the hidden image (Figure 3). Each gray circle in the image represents a tumor in a patient. All objects in the hidden image vary in size, shape and quantity. The two possible shapes are circle and triangle; the quantity and size of each object is proportional to the size of the canvas. Every time the player successfully deduces the correct number of the gray circles in a level, they progress to the next level. As the player progresses, the general difficulty of the program should increase. However, if the player fails to identify the correct number of gray circles in the image, they are returned to a lower level.

By default, the background color is black; however, once the player reaches level 8, all levels ending with the digit 8 are “inverted” (i.e. a white background with black whole-tone objects). Additionally, past level 8, there is a 1/6 chance that any level can be inverted.

To further vary the difficulty of each level, we alter the number of pixels in the hidden image. Each dimension (height and width) of the hidden image is calculated/generated separately. The base size of the canvas is 256x256. Each dimension can be randomly increased by 128 pixels for every 5 levels. For example, at levels 5–9, the possible canvas sizes are: 256x256, 256x384, 384x256, and 384x384, and at levels 10–14 the possible canvas sizes are: 256x256, 256x384, 256x512, 384x256, 384x384, 384x512, 512x256, 512x384, and 512x512. In addition, because the number of objects is influenced by the size of canvas (via a random number generator), larger canvases may contain more objects to find.

While the number of levels currently possible is over 2 million (2^32^ – 1), most players will likely lose, give up, or get bored or die before they reach this level. Additionally, the practical limit of the game depends on the speed and memory of the computer the player is playing on. The code has been optimized to try to minimize the demand on the CPU, as the CPU speed seemed to be the weakest link in most situations.

Variations could be readily incorporated in the program. For example, the hidden image could be a real CT slice with real or simulated tumors in it.

#### Analysis/purpose of CT brush gameplay

The CT Brush game has a tracking feature, which causes the program to pipe specific data to a TCP/IP connection [74]. The data tracked is comprised of: the current level the player is working on, the hidden image for that given level, and the player’s brush movements and refine requests. This data may be analyzed later to deduce patterns in the user’s approach, which could help improve CT algorithm design. We thus hope to find strategies which hone in on features of the image that result in a more dose-efficient detection of tumors. If any of the player strategies could be ascertained and formalized into a computer algorithm, then CT Brush algorithms could be automated and used to run hardware CT scanners. An automated CT Brush is, in effect, an intelligent flying spot 3D CT [17,75].

Thus, the advantage of CT Brush is that it allows us to explore inside a patient, attempting to hone in on the image information, while trying to keep total x-ray dose to a minimum, perhaps at substantially less than the dose of shotgun CT imaging. An analogy can be made to eye tracking of radiologists, in which the direction of their gaze is recorded [76-78]. The difference here is that the image data is acquired as the gaze is changed. In fact, eye tracking glasses or computer cameras [79] could be used to run the CT brush.

**Figure 4:**
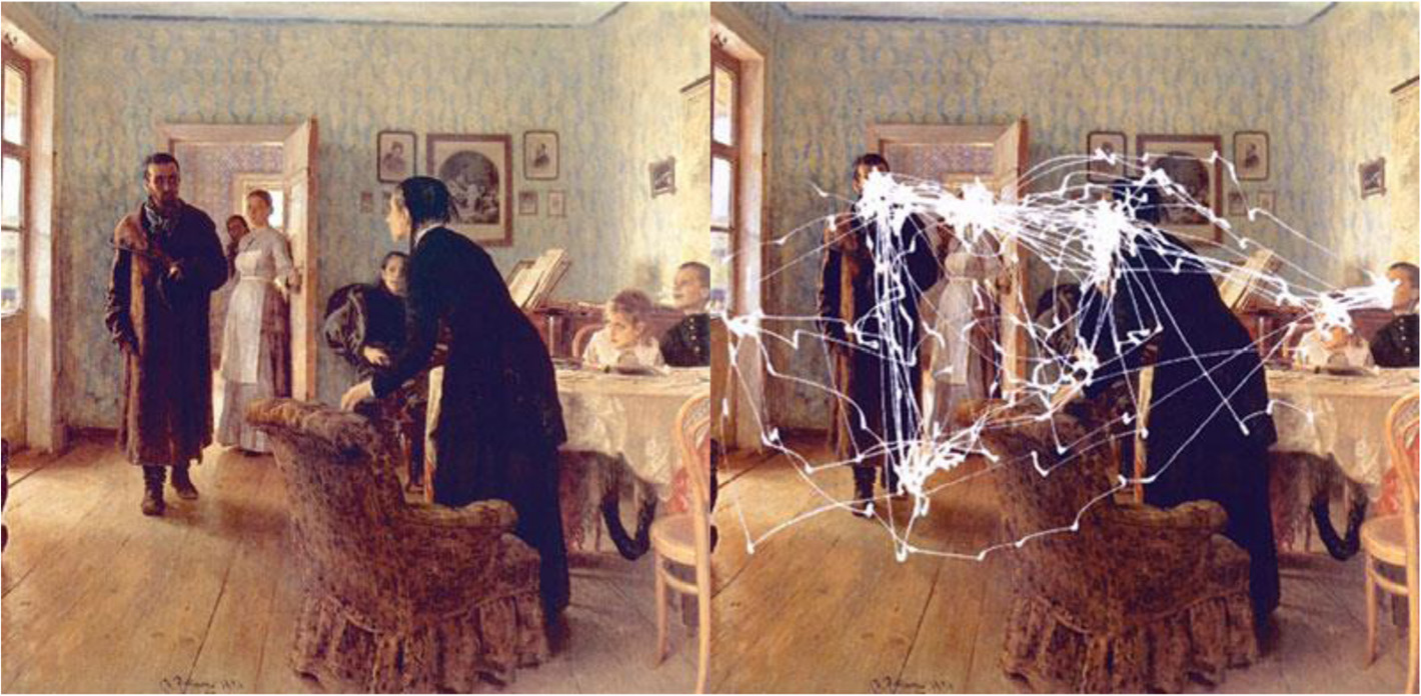
An eye tracking experiment by Alfred L. Yarbus [150] in which the eye movements have been superimposed [84] on a painting "Unexpected Visitors" by the 19th Century Russian artist Ilya Repin. The eye tracking is easier to appreciate if the two images are viewed as a stereo pair [151]. Figure 5 *Middle* and *Right* may also be viewed in stereo.

**Figure 5:**
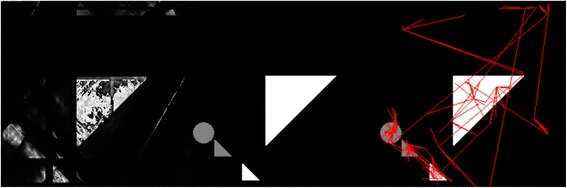
*Left*: Same picture as Figure 3 *Left* (except that the game has been played a bit further), showing the reconstruction at this early stage of the game, without the star of rays. *Middle*: The hidden image containing one target “tumor” (gray circle) and the cluttering objects near and far. This image was hidden from the player during gameplay. *Right*: The track of the central-points used in the game by the player, superimposed on the hidden image, which the player couldn’t see. The long straight lines are hand/eye movements that were straightened by the mouse interpolation algorithm. While the image on the left includes the target, it does not accurately reconstruct any of the objects, and some artifacts appear that are comparable to the real objects. As play continues, these artifacts can be seen to have disappeared (Figure 3, *Middle* and *Right*), perhaps because the player paid some attention to them (*Right*).

As a step towards analysis of players’ actions, we may consider a process analogous to eye tracking of a scene, such as the study depicted in Figure 4. The main difference is that the scene is initially invisible (Figure 1). The hand/eye tracking by the user for the game played in Figure 3 is shown in Figure 5. This visual approach may permit us to use methods developed in studies of visual behavior [78,80-83] to learn what the player pays attention to as he/she reconstructs the scene, such as in the construction of a “story board” from gaze tracking [84-86]. It is plausible that displaying the history of her/his hand/eye tracking might aid a player in deciding on future moves. This feedback, an on the fly version of the hand/eye tracking as in Figure 5*Right*, could readily be added to the CT Brush game.

#### CT brush mathematics

Any CT reconstruction is generally one possible solution to the equations describing the relationship between raysums and the pixels or voxels of the image. Even with cross sectional images in 2D we are dealing with a slab of voxels. So, in this section of the manuscript, we will always refer to the image elements as voxels rather than pixels. The generalization from 2D to 3D is then conceptually straightforward. As we usually deal with many more unknowns (voxels) than measurements (raysums), there is a whole hyperspace of possible solutions. Most CT algorithms generate a single reconstruction from this vast array of possibilities.

In previous work we showed how one could take an intelligent “walk” in the space of solutions, and explore for the existence of substantially different solutions to the same CT equations [73]. This “walk” was a “clunky” approach, in that one had to design “objects” to be added to the image, or subtract objects in the image, and then let convergence of the iterative equations take one back to the solution hyperspace. The CT brush is much easier to use as an exploratory tool. Pointing at and brushing over an area of possible interest is a much more natural operation. A given area can be “scrubbed” with a CT brush until it is apparent that something interesting lies there or not. If an edge shows up, the brush can be moved along the edge, to follow its trajectory in the image. If the brush width is varied, the operation can be sped up with a wider brush, or small details can be tested by using a finer brush.

To simulate this process in 2D, we begin with a square image *U*(*i*,*j*),*i*,*j* = 1,…,*N* which is stored in the computer, but is not seen by the player (“hidden” or “unknown” to the player). This could represent a cross section of a patient. The image is kept hidden from the player, because in real life we would not have that image, and we would have to decide how to collect the data to get just enough image detail to decide on tumor detection.

We approximate the *R* rays through a point in the image by a binary (0 or 1 valued) mask *M*(*k*,*l*,*r*),*k*,*l* = −*N*,,,*N*;*r* = 1,…,*R*. The dimensions of *M* are chosen so that if its center at (*k*,*l*) = (0,0) is placed over any voxel (*a*,*b*) in *U*, the mask will completely cover *U. M* is actually a stack of *R* masks, one for each ray *r*, because separate data is available for each ray traversing the image. Mathematically, by using a binary mask we avoid the problem and the computing time of calculating the “weight” to be assigned to each pixel in a given ray [31]. In the software, however, we used a staircase function, representing a binary ray-line (i.e., it is not anti-aliased), so that is not actually stored. The mask summed over its rays is in effect a thresholded version of the point spread function of an ART-type CT algorithm [63,65,67].

The raysum for a given ray *r* through point (*a*,*b*) in *U* may now be written as:

1 $$ S\left(a,b,r\right)={\displaystyle \sum_{k=-N}^N{\displaystyle \sum_{l=-N}^NM\left(a-k,b-l,r\right)}}U\left(a,b\right) $$

This formulation allows *M* to represent any kind of ray, including parallel, diverging fan or cone beam, or converging [17,87]. We are assuming that the rays available from the x-ray sources come in parallel bundles with uniform properties, so that the same mask *M* may be used for all voxels. Put another way, *M* is translationally symmetric, i.e., spatially homogeneous. Any (*x*,*y*,*z*) mechanical scan mode would fit these constraints; however, these constraints could be lifted.

We start with an initial image *A*_0_(*a*,*b*) = 1;*a*,*b* = 1,…,*N*, which would typically be a uniform image. Each time a ray is processed, or some other image processing operation is performed, we increment the index on *A*. The general gameplay would consist of “mouse down”, drag, and then “mouse up”, resulting in a sequence of voxels (*a*_*i*_,*b*_*i*_),*i* = 1,…,*m* representing the central points of the CT brush stars by which the player irradiated the image. These voxels are recorded via the tracking system. If the player puts the mouse down with a canvas image *A*_*s*_, then the sequence of images up to *A*_*s+m*_ would be generated and displayed.

Since we are dealing with the rays one by one, we may use the general ART-type [30] computed tomography algorithm. The specific algorithm used to adjust the values of the pixels along a ray may be additive ART [30], multiplicative ART (MART) [30,88-91], streak suppression ART [39], or any other variant on this theme. In our implementation of 2D CT Brush, we used MART, and considered each previously unused ray of the CT brush in a clockwise order from horizontal:

1. Calculate the raysum for the ray (*a*,*b*,*r*) by traversing the hidden image *U*:
  2 $$ S\left(a,b,r\right)={\displaystyle \sum_{k=-N}^N{\displaystyle \sum_{l=-N}^NM\left(a-k,b-l,r\right)}}U\left(a,b\right) $$
2. Calculate the current estimate of the raysum for the ray (*a*,*b*,*r*) by traversing the current estimate for the image *A*_*i*_:
  3 $$ {S}_i\left(a,b,r\right)={\displaystyle \sum_{k=-N}^N{\displaystyle \sum_{l=-N}^NM\left(a-k,b-l,r\right)}}{A}_i\left(a,b\right) $$
3. Find new values for each voxel in the ray, represented by *M*(*a*-*k*,*b*-*l*,*r*)=1, such that: *S*_*i* + 1_(*a*, *b*, *r*) = *S*(*a*, *b*, *r*). This step may be done differently based on the flavor of ART. In our implementation, multiplicative ART (MART) was used. Therefore, we used the formula:
  4 $$ {A}_{i+1}\left(a,b\right)=\frac{S\left(a,b,r\right){A}_i\left(a,b\right)M\left(a-k,b-l,r\right)}{S_i\left(a,b,r\right)} $$
4. When the values of voxels along a ray are changed, so are the raysums for all of the previously used rays that intersect the voxel. Therefore, at each step, or periodically, the previously used rays would also need to be adjusted per the CT algorithm. Such adjustments can be made iteratively until a convergence criterion is satisfied. With all these voxels updated, we have the next image *A*_*i*+1_. Clicking the “Refine” button will perform one iteration of refinement, in our program. Thus, we track the number of refinements the player uses. The player sees the results of each iteration, and can judge if further iterations are warranted.

Since the MART algorithm always leaves a raysum of zero as zero (Equation 4), the initial image, while uniform, should not contain zeros in the region of interest. Thus we set *A*_0_(*a*, *b*) = 1 ∀ (*a*, *b*). All of the equations in this section are implemented in the Java method *do_projection*, which is located in the file path *src/org/alvaregordon/ctbrush/GFXMath.java* in the appended software.

CT Brush could have been implemented by considering all of the rays through the central point at once as in SIRT/SART (Simultaneous Iterative/Algebraic Reconstruction Techniques) and its variants [70,92-97], or with variants on the ART algorithm itself [39,89,90,98-101], including parallel computing versions [91,102,103].

#### Estimation of the reduction in dose with CT brush

**Figure 6:**
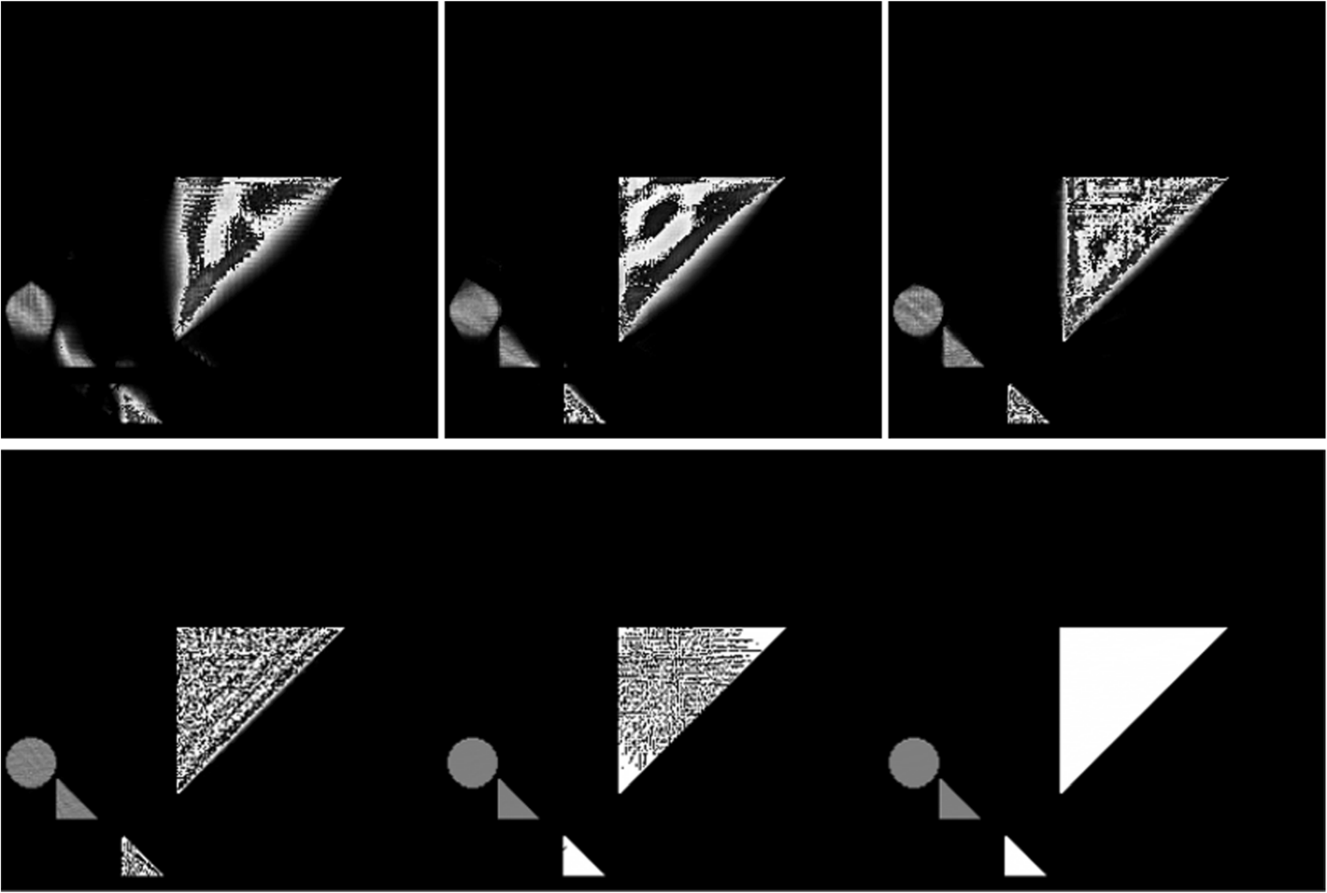
A sequence of MART reconstructions of Figure 5 *Middle*, with increasing numbers of views: 5, 6, 9, 18, 36, and 72, equally spaced in angle. These were generated by using the CT Brush code, looping the central-point of the CT brush through all of the pixels (each initially set to 1), and refining until convergence. Such “traditional” CT images provide a visual comparison with the player driven, object dependent CT Brush reconstructions in Figure 3. Of course they have more uniform spatial resolution, as the point spread function is approximately spatially homogeneous [63,65,67].

**Figure 7:**
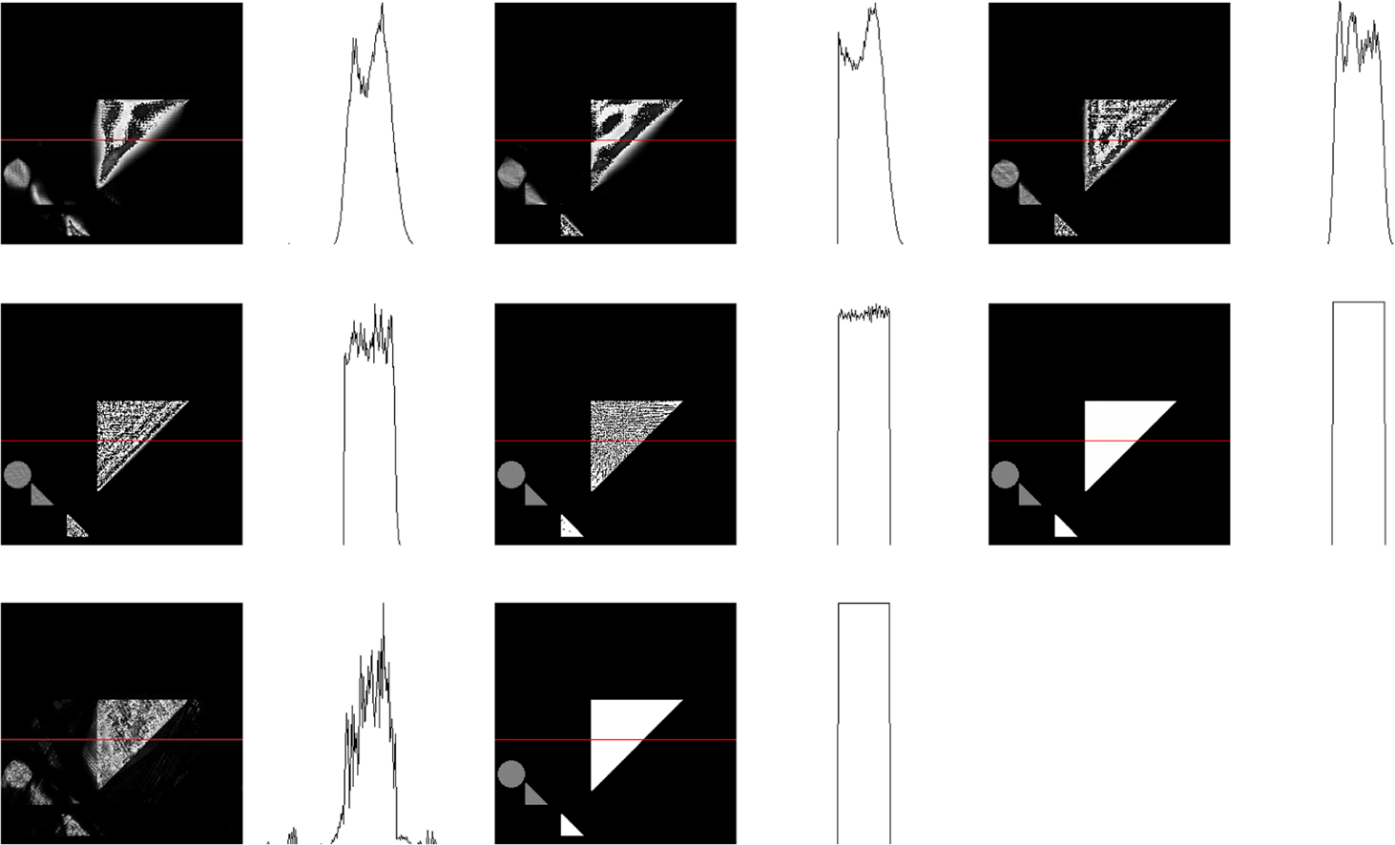
The top two rows (3 columns per row) contain the line profile plots for each of the line constructions in Figure 6: 5, 6, 9, 18, 36, and 72 view MART reconstructions, respectively. The bottom row (2 columns) contains the following line profiles: the line profile for an example of manual play on the left, and the line plot for the hidden image on the right. All line profiles are sampled horizontally at the y-coordinate 145 of these 256x256 pixel images.

For visual comparison of Figures 3 and 5 with “traditional” CT algorithms and use of rays, in Figure 6 we show the reconstruction of the image (Figure 5*Middle*) that was unknown to the player, as reconstructed by MART with increasing numbers of parallel projections equally spaced in angle. In Figure 7 we show line profiles [104], which give another visual comparison.

For dose comparison, we need to find a common basis for comparing the irregular usage of rays in CT Brush with traditional MART. While, of course, absorbed dose is most important to patients, that is an object dependent measure. We thus decided to use a simpler parameter, i.e., the number of unique rays. Since in CT Brush each distinct ray is used only once, corresponding to pointing an x-ray microbeam in a given direction, this count would seem to be a good measure of the total emitted dose the patient is subjected to (Table 1).

**Table 1:** **Comparison of the relative emitted dose for all of the standard CT images generated in Figure** **6** **compared to the manual CT Brush play of Figure** **3** ***Right***

**R = # of views per star**	**Angle between rays**	**Total # of unique rays** ***T*** **(Eq.** 5 **)**	**Effective # of rays** ***E*** **(Eq.** 8 **)**	**Manual/** ***T***	**Manual/** ***E***
				**(%)**	**(%)**
manual play	-	2,126	-	-	-
5	36°	262,400	1,618	0.81	131.4
6	30°	327,936	1,913	0.65	111.1
9	20°	524,544	2,930	0.41	72.6
18	10°	1,114,368	5,861	0.19	36.3
36	5°	2,294,016	11,747	0.093	18.1
72	2.5°	4,653,312	23,606	0.046	9.0
180	1°	11,731,200	58,771	0.018	3.6

All of these angles have irrational tangents. The stars used here all include the 0° ray, whose tangent is rational, so that Equation 5 is used for *T*.

**Figure 8:**
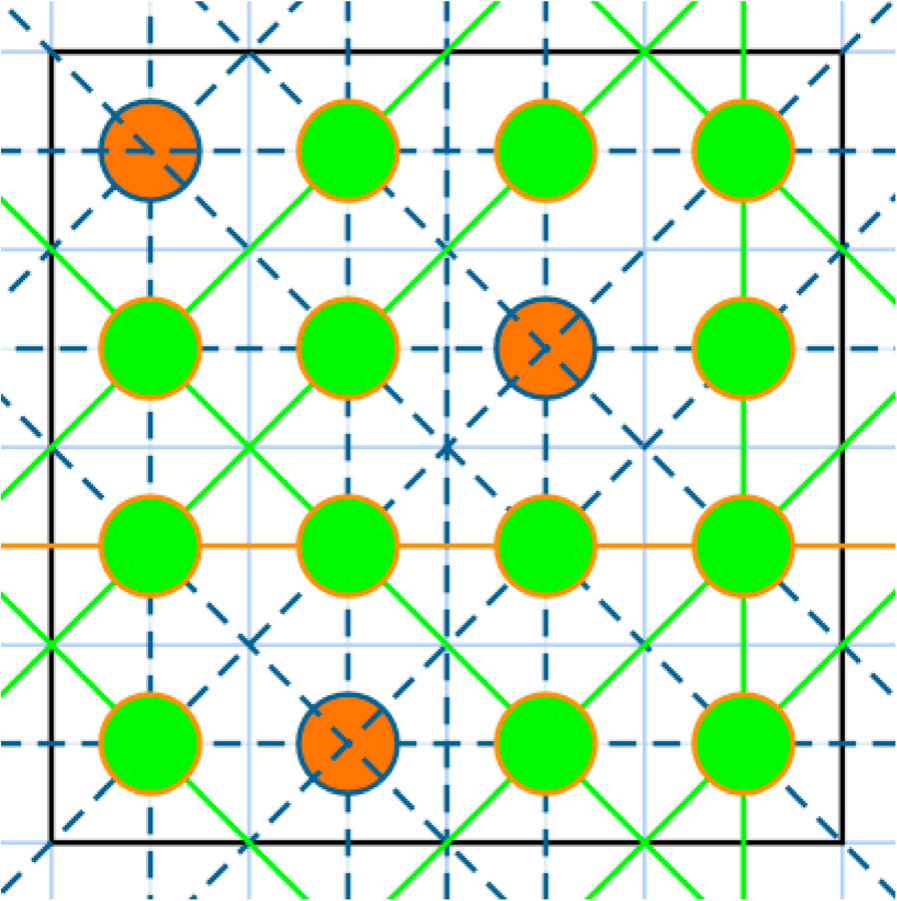
The problem of counting the unique rays for a given number of views is illustrated here on an *N* × *N* = 4 × 4 picture with *R* = 4 views with rational tangents. We were able to place only 3 stars so that none of their rays overlapped. Their centers are shown as red circles and their unique rays as dashed lines. The missing rays are drawn as solid green lines. For the 16 pixels shown here, the total number of unique rays is therefore *T* = 3*R* + 8 = 20. This is substantially less than *T* = *RN* ^2^ = 4 × 16 = 64. Thus 31.25% of the possible rays with this star are unique.

**Figure 9:**
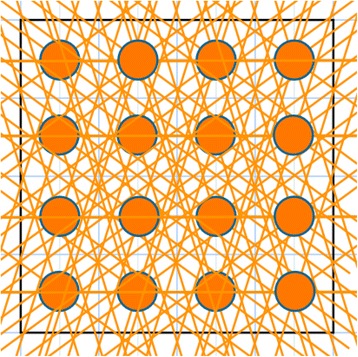
Here we show all of the stars through all 16 pixels of the same array as Figure 8, but with 5 evenly spaced views for which the tangent is irrational, except for 0°. The total number of unique rays is thus 68. With a slight rotation all of the rays could have irrational tangents, raising the number of unique rays to *T* = *MN* ^2^ = 5 × 16 = 80. Thus 85% of the possible rays with this star are unique, and with the rotation this would rise to 100%. Of course, with truncation of rays to staircase functions, as we have done for the sake of computational speed, many of the otherwise distinct rays end up with the same staircase function. This would not be the case if weights between 0 and 1, rather than 0 or 1 only, were used [31].

However, two problems became apparent. As shown in Figure 8, if the star angles have rational tangents, many rays overlap, and since each should not be counted more than once, this leads to a difficult counting problem. On the other hand, if we deliberately use star angles with irrational tangents, the overlap problem is reduced or eliminated (Figure 9), and we obtain:

5 $$ T=R{N}^2-N\left(N-1\right) $$

The negative term is due to the 0° ray, whose tangent is rational. With a slight rotation of all the angles, their tangents could all be irrational, so that:

6 $$ T=R{N}^2 $$

The number of unique rays far exceeds what is reasonable for a digitized picture because we represent each ray by a staircase function, and many rays will have the same or very similar staircase approximations. However, since we used stars going through every pixel to emulate the standard MART algorithm, we indeed used the whole set of these staircase functions *M*, so the result is better than we might otherwise anticipate from MART. Furthermore, here we are using pseudoprojections [94,105,106], i.e., raysums calculated from an already digitized image *U*, which make a small difference from real world data.

Another way to estimate the number of rays is to pretend that all of them at a given angle are equally spaced, with a width equal to the pixel width. These are not the rays used by CT Brush. While this would be the ordinary way of coding MART, it in general involves specifying weights [31], and thus comparison with CT Brush as implemented here would be problematic. A bit of trigonometry shows that for rays at angle *θ* the number of rays intersecting an *N* × *N* square is:

7 $$ {N}_{\theta }=N\left(1+\left| \tan \theta \right|\right)\left| \cos \theta \right| $$

and the emitted dose becomes:

8 $$ E={\displaystyle \sum_{r=1}^R{N}_{\theta (r)}} $$

Figure 3*Right* was reconstructed by playing CT Brush accumulating 2,126 rays, as calculated by the method shown in Figure 8. In retrospect the user started with 3 views, then refined with 4, 5 and 8 views, a mixture of angles with irrational and rational tangents. We can see in Table 1 that the bounds formed by *E* and *T* compared to this single example of CT Brush play are not tight, but do suggest that substantial dose reduction is achievable via human computing.

#### Mouse interpolation

On the computer we used (2008 Apple MacPro – Two Quad-Core 2.8 GHz Intel Xeon Processors, 20GB RAM, ATI Radeon HD 2600XT Graphics Card, and OS X 10.6.3 Server), we measured the mouse sampling rate as one sample per 16.5 msec. This meant that pixel locations, read via the mouse, would not be consecutive neighbors. Thus, mouse interpolation was required. For this, we used linear interpolation [107].

#### CancerZap!, a first person shooter game

**Figure 10:**
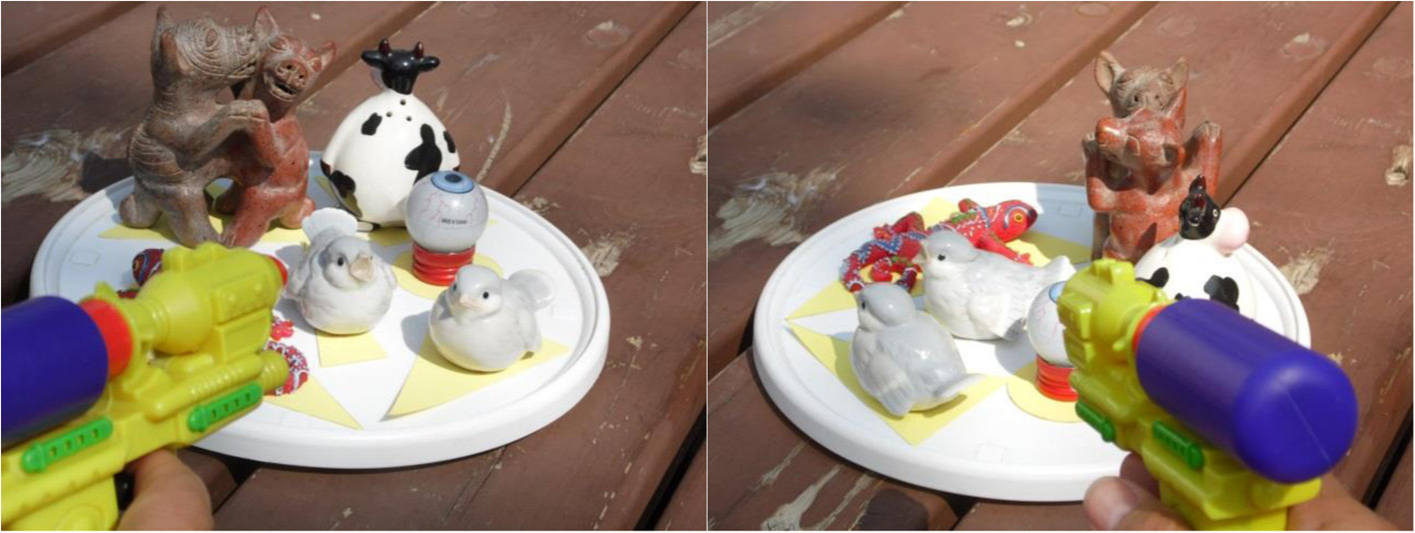
A mockup of what the screen might look like in a CancerZap! video game. The player shoots x-ray photons at a scene that is rotating, with one object (the Martian eye [152]) in this case representing the “bad guy” tumor. The gun’s lateral motion would correspond to a fan beam. As in CT Brush, the objects would only become visible as they were shot at, accumulating x-ray dose. In the plane, the ornate figures would be represented by simpler “footprints”, such as triangles and circles, shown here beneath them.

CT Brush proved a bit too abstract for children, inspiring us to think about an alternative, shoot ‘em up game. This would differ from CT Brush in the following aspects:

1. What is seen in the front of the screen is a machine gun pointing away that shoots lots of individual x-ray photons (Figure 10).
2. The gun swivels, so that it is creating, in effect, a fan beam.
3. The good and bad guys (normal and tumor tissues) are represented by lively action figures standing on a platform, so there is a 3D scene to shoot at.
4. The platform keeps rotating like a merry-go-round, though perhaps reversing and changing speed at random, providing moving targets, even though the action figures don’t move across the platform. This simulates multiple views.
5. The player has to identify which figures are the bad guys. They might grimace, wear weird clothes, collapse dead when shot too much, revealing their identification, etc.
6. The image being reconstructed is in some way a vertical projection of the action figures onto the platform.

CancerZap! would allow us to explore intelligently steered dose reduction for photon limited CT imaging, where Poisson noise effects are huge [17,108-115]. So it would not be just for children.

## Results and discussion

Our purpose here is to place the CT Brush game into the public domain, so that experimentation can begin in developing object dependent strategies for x-ray dose reduction in CT. Various problems in science are being solved via crowdsourcing by taking advantage of human intuition [116-126]. Some of these are games that are explicitly used in “human computing” for labelling images [127] or finding objects in images (that are not hidden from the player, as here) [128]. We anticipate feedback well beyond our own limited imaginations, regarding this as a communal effort. By making it fun and straightforward, we hope that all kinds of people, lay to professionals, will contribute to the goal of x-ray CT dose reduction.

Our hope is that a combination of fun and altruism will draw people in to play the game. We collected some qualitative responses from a small group, prior to writing the article, and the consensus was that the game was fun to play. We hope this enjoyment will scale well to a larger audience. We also hope that an altruistic attitude towards advancing science will draw some people towards playing the game. In addition, because the program is open-source, we welcome other developers to modify our source code and create fun game “mods”.

### Future improvements

CT Brush is available online, with open code, permitting its further development [129], and we hope to get feedback from readers and players. Here are some examples for improvement of the CT Brush game:

1. The CT brush could be used sparingly, by reducing its x-ray intensity, thus permitting a rough sketch of the patient at low dose, analogous to a scout scan [130]. This would, of course, have to be a noisy image, but if any feature caught the eye, it could be run over again with the CT brush set to a higher intensity. A suspect region would then be sharpened up, or, alternatively, smoothed out, depending on whether the apparent feature was really present or just due to noise fluctuations.
2. Different weights could be assigned to rays in different directions, to allow following of edges, etc. The direction of mouse movement could be used to automatically adjust these weights. This approximates linear receptive fields in vision [131,132].
3. Rays could be anti-aliased.
4. CT Brush could be implemented with consideration of the order in which rays are analyzed: ART algorithms converge most rapidly if the rays are considered in a particular order, where consecutively processed rays are as close to mutually perpendicular as possible [133]. The primary impact of this would be to reduce the number of Refine steps to convergence.
5. Local dose could be kept under a given maximum, to avoid radiation burning, by locking out rays that would cause the maximum to be crossed. Regions in which the local dose had reached its maximum could be displayed to the player.
6. A palette of image processing operations [134] could be made available that could, for instance, sharpen or smooth the image, apply various norms [135], create pseudocolors, round up localized pixels into compact structures, apply histogram equalization, fit models to the image [136,137], sketch in guesses for tumors based on hints in the image at a given stage, erase suspected artifacts, etc. [73]. By hitting “Refine” a few times, the altered image would be made consistent with the raysum data. This involves no cost in dose, yet allows the player to bring in many kinds of *a priori* information.
7. In cases where the total angle range for the rays may be restricted [72], as in breast CT [69,138-140] or electron or visible light microscopy [141-143], deconvolution of the point spread function [62,63,65,67,144] corresponding to the CT brush could be invoked, again with no cost in dose. Ringing artifacts could be damped by filtering and/or iteratively applying “Refine”.
8. Cumulative dose used so far could be compared to the best (lowest) score obtained by any player so far, via the Internet.
9. A multiuser version could allow players to compete in finding the first or all of the tumors present, to challenge one another with different hidden images, etc.
10. A library of realistic hidden images could be developed for radiologists to hone their skills at detecting tumors inside various tissues at low dose, with the tumors being real or simulated [145].
11. The hand/eye tracking could be made visible to the player, as a guide to what moves they have already tried. Additional information about the tracking could also be visualized: the number of rays in the star, number of rays of the star not previously used, ray width, and time stamps.
12. Compressive sensing (CS) algorithms for CT could be included as part of CT Brush.

We can elaborate on the use of compressive sensing in CT Brush, as follows. In general, the CT reconstruction problem can be formulated, in the noise free case, as finding a solution to a matrix:

9 $$ Ax=b $$

where *x* is a vector representing the unknown image, *A* is a sparse measurement matrix, and *b* is the data (raysums). Here we are using the notation and vocabulary of recent papers on CS in CT [28,29]. In particular, CS has been applied to a set of rays selected randomly and independently of the image (using the commercial optimization software MOSEK [146]). Not surprisingly, performance was worse than with uniformly selected rays [147]. The situation is a bit different in CT Brush, because the rays are selected by the player in an image dependent manner. This means that with the addition of each ray by the player, the matrix *A* and the data *b* are changed, and all of the components of *x* (the pixels or voxels) have to be recalculated. Instead of applying the MART algorithm, which is ray based and therefore simple to implement with deferred refining, we could consider solving a separate global CS problem at each step:

10 $$ {A}_i{x}_i={b}_i,i=1,2,3,\dots {N}_{tumor(s) found} $$

The index *i* refers to the rays consecutively added by the player. This “progressive compressive sensing” algorithm could be attempted in future work, addressing three questions: 1) can the CS software be run fast enough to keep up with the hand/eye motion of the player? 2) does CS reduce the total number of rays needed for the player to reach a decision, *N*_*tumor(s)found*_? 3) Will image-dependent dictionaries [148,149] lead to further CS improvement in the image? CS CT via Equation 10 has the advantage over MART (Equation 4) of calculating values for all the pixels, whereas MART only calculates values for pixels along the rays used so far, but that very fact also slows the CS computation in comparison with MART.

With open source code, alternative CT algorithms to MART, including CS, may be added by participating programmers. The experience gained from many people playing CT Brush should improve our concept of how to develop CancerZap!.

In previous work [73] we showed how an intelligent walk in the hyperplane of solutions to a set of underdetermined CT equations allows one to hone in on the structures present in the unknown image. Now that computers are so much faster than in 1973, it becomes practical to explore similar ideas in real time games. As CT dose has become a major issue in radiology, we hope that these games will lead to intelligent algorithms and new designs for CT scanners that reduce the dose to the minimum for the screening or diagnostic task at hand.

## Conclusions

Nonlinear and object dependent algorithms abound in computed tomography. Compressive sensing is beginning to show how to best use linear methods. However, all of these depend on spraying the patient with x-ray photons. The major improvement in gaming CT Brush and CancerZap! approaches to CT algorithms is that the very act of aiming the x-ray beams becomes object dependent. This is why we suggest that these games point a way to significantly greater dose reduction in x-ray CT. Preliminary results of playing CT Brush suggest that emitted dose might be reducible by a factor of 2 to 10 compared to current practice.

### Availability and requirements

- ○ Project name: CT Brush
- ○ Project home page: http://home.cc.umanitoba.ca/~alvare/ctbrush
- ○ Operating system(s): Platform independent
- ○ Programming language: Java
- ○ Other requirements: Java 1.5 or higher
- ○ License: Creative Commons 3.0 by-sa
- ○ Any restrictions to use by non-academics: none

The file *ctbrush.jar* is the Java JAR archive for the CT Brush game. This file may be used to run the Java applet. Currently, the only parameters available to the applet are the track parameter and the port parameter. Each of these parameters is optional. If the track parameter is set to anything, other than blank, the CT Brush applet will track the player’s brush strokes and progress. By default, this tracking information will be sent to port 4444. However, an alternate port may be specified, by passing a “port” parameter to the applet.

The tracking information is represented as “pseudo-functions”, where only one function is allowed on each line. Each level is preceded by a line containing ten (10) equal signs (‘=’). In addition, the triangles and circles describing a level are preceded, on each line, by four (4) space characters.

Each pseudo-function is followed by parentheses. Inside the parentheses, parameters may be passed. These parameters are generally numbers, which are represented in the table below by the number sign (‘#’); however, some of the pseudo-functions also pass boolean values (denoted as ‘bool’) as parameters:

- *level*(#:#:#) – indicates that the player has progressed to a new level. The first number passed is the level number, the second number is the width of the level canvas, and the third is the height of the level canvas.
- *t*(#,#,#:*bool*) – indicates where a triangle is located within the current level canvas. The first 2 numbers are the x and y coordinates of the triangle. The next number is the size of the triangle. Because all of the triangles are right-angle isoceles triangles (with two 45 degree angles and one 90 degree angle), the size corresponds to either of the non-hypotenuse sides. Lastly, the boolean corresponds to whether the triangle is gray half-tone (true) or full tone (false).
- *c*(#,#,#:*bool*) – indicates where a circle is located within the current level canvas. The first 2 numbers are the x and y coordinates of the circle. The next number is the radius of the circle. Lastly, the boolean corresponds to whether the circle is a gray half-tone (true) or full tone (false).
- *m*(#,#:#^#_#) – indicates a player mouse-brush movement within the level. The first two numbers, from the left, are the X and Y-coordinates, respectively; the third number corresponds to the width of the brush; the fourth number corresponds to the number of rays in the brush; the right-most number corresponds to the rotation of the brush. Currently, brush rotation is not implemented; however, it may be easily added in future versions.
- *r*() – indicates the player has chosen to perform a refinement action.
- *g*(#) – indicates the player has finished the level, and guessed the number of gray circles. The number passed by this pseudo-function is the player’s guess.

The file *ctbrush.*zip is a zip file that contains all of the Java source code for CT Brush. The source code files are located in the “src” directory. The zip file also contains some optional files and directories, to assist users with editing and building the code: the files “build.xml” and “app.properties” may be used to build the CT Brush project using Apache Ants; the directory “nbproject” may be used to open the CT Brush code with NetBeans.

The file *ctdocs.zip* is a zip file that contains all of the JavaDoc API documentation for the CT Brush project. All of the JavaDoc API documentation is in HTML format. To view this documentation, please load index.html (contained within this file) into a web-browser.

## Additional files

Additional file 1:
**The file ctbrush.jar is the Java JAR archive for the CT Brush game.** This file may be used to run the Java applet. Currently, the only parameters available to the applet are the track parameter and the port parameter. Each of these parameters is optional. If the track parameter is set to anything, other than blank, the CT Brush applet will track the player’s brush strokes and progress. By default, this tracking information will be sent to port 4444. However, an alternate port may be specified, by passing a “port” parameter to the applet. Additional file 2:
**The file ctbrush.zip is a zip file that contains all of the Java source code for CT Brush.** The source code files are located in the “src” directory. The zip file also contains some optional files and directories, to assist users with editing and building the code: the files “build.xml” and “app.properties” may be used to build the CT Brush project using Apache Ants; the directory “nbproject” may be used to open the CT Brush code with NetBeans. Additional file 3:
**The file ctdocs.zip is a zip file that contains all of the JavaDoc API documentation for the CT Brush project.** All of the JavaDoc API documentation is in HTML format. To view this documentation, please load index.html (contained within this file) into a web-browser.

## Abbreviations

ART: Algebraic reconstruction technique
CPU: Central processing unit
CS: Compressive sensing
CT: Computed tomography
MART: Multiplicative algebraic reconstruction technique
RAM: Random access memory
SIRT: Simultaneous iterative reconstruction technique
TCP/IP: Transmission Control Protocol/Internet Protocol
XLCT: X-ray Luminescence CT

## References

[1] Renold M. MyPaint: Create your own brush. 2005. [http://mypaint.intilinux.com/?page_id=173]

[2] SourceForge. Qaquarelle. 2013. [http://sourceforge.net/projects/qaquarelle/]

[3] SourceForge. DrawPile. 2014. [http://sourceforge.net/projects/drawpile/]

[4] FlowPaint. FlowPaint. 2014. [http://www.flowpaint.org/]

[5] Krita Foundation. Krita: Open Source Software for Concept Artists, Digital Painters, and Illustrators. 2014. [https://krita.org/]

[6] Microsoft. Paint. 2014. [http://windows.microsoft.com/en-ca/windows7/products/features/paint]

[7] Gordon R, Colquhoun GD (2012). CancerZap!: Battleship meets Where’s Waldo?. BioPhotonics.

[8] Gordon R, Sivaramakrishna R (1999). Mammograms are Waldograms: why we need 3D longitudinal breast screening guest editorial]. Appl Radiol.

[9] Wikipedia. Where's Wally? 2014. [http://en.wikipedia.org/wiki/Where%E2%80%99s_Waldo]

[10] Resnick BJ (2007). Battleship - A senior design preparatory experience. Proceedings 2007 37th Annual Frontiers in Education Conference, Global Engineering: Knowledge without Borders - Opportunities without Passports.

[11] Wikipedia. Battleship (game). 2012. [http://en.wikipedia.org/wiki/Battleship_(game)]

[12] Von Wickler C. Battleship (1931). 2012. [http://www.boardgamegeek.com/boardgame/2425/battleship]

[13] Port AC, Yampolskiy RV, Mehdi Q, Elmaghraby A, Marshall I, Moreton R, Ragade R, Zapirain BG, Chariker J, ElSaid M, Yampolskiy R, Zhigiang NL (2012). Using a GA and Wisdom of Artificial Crowds to solve solitaire Battleship puzzles. 2012 17th International Conference on Computer Games (CGAMES), Louisville, Kentucky, July 30-August1, 2012.

[14] Learn4Good Ltd. Fun Online Games for Kids: Battleship. 2012. [http://www.learn4good.com/games/board/battleship.htm]

[15] Wikipedia. Video game genres. 2014. [http://en.wikipedia.org/wiki/Video_game_genres]

[16] Badea CT, Stanton IN, Johnston SM, Johnson GA, Therien MJ (2012). Investigations on X-ray luminescence CT for small animal imaging. Proc SPIE.

[17] Gordon R, Tot T (2011). Stop breast cancer now! Imagining imaging pathways towards search, destroy, cure and watchful waiting of premetastasis breast cancer. Breast Cancer - A Lobar Disease.

[18] Wikipedia. Golf: Scoring. 2014. [http://en.wikipedia.org/wiki/Golf_-_Scoring_and_handicapping]

[19] Zhao YZ, Brun E, Coan P, Huang ZF, Sztrókay A, Diemoz PC (2012). High-resolution, low-dose phase contrast X-ray tomography for 3D diagnosis of human breast cancers. Proc Natl Acad Sci U S A.

[20] Wang Z, Gao K, Ge X, Wu Z, Chen H, Wang S (2013). X-ray phase radiography and tomography with grating interferometry and the reverse projection technique. J Phys D Appl Phys.

[21] Olivo A, Gkoumas S, Endrizzi M, Hagen CK, Szafraniec MB, Diemoz PC (2013). Low-dose phase contrast mammography with conventional x-ray sources. Med Phys.

[22] Starck JL, Murtagh F, Fadili JM (2010). Sparse Image and Signal Processing: Wavelets, Curvelets, Morphological Diversity.

[23] Chen GH, Tang J, Nett B, Qi ZH, Leng SA, Szczykutowicz T (2010). Prior Image Constrained Compressed Sensing (PICCS) and applications in x-ray computed tomography. Curr Med Imaging Rev.

[24] Kaganovsky Y, Li D, Holmgren A, Jeon H, MacCabe KP, Politte DG (2014). Compressed sampling strategies for tomography. J Opt Soc Am A Opt Image Sci Vis..

[25] Hu Z, Liang D, Xia D, Zheng H (2014). Compressive sampling in computed tomography: Method and application. Nucl Instrum Methods Phys Res Section A-Accelerators Spectrometers Detectors and Associated Equipment.

[26] Saha S, Tahtali M, Lambert A, Pickering M (2013). Compressed sensing inspired rapid Algebraic Reconstruction Technique for computed tomography. 2013 IEEE International Symposium on Signal Processing and Information Technology (ISSPIT), 12-15 Dec 2013.

[27] Langet H, Riddell C, Trousset Y, Tenenhaus A, Lahalle E, Fleury G (2011). Compressed sensing based 3D tomographic reconstruction for rotational angiography. Lect Notes Comput Sci.

[28] Jørgensen JS, Kruschel C, Lorenz DA. Testable uniqueness conditions for empirical assessment of undersampling levels in total variation-regularized x-ray CT. Inverse Problems in Science and Engineering. 2014. doi:10.1080/17415977.2014.986724.

[29] Jørgensen JS, Sidky EY, Hansen PC, Pan X (2014). Empirical average-case relation between undersampling and sparsity in x-ray CT. Inverse Probl Imaging.

[30] Gordon R, Bender R, Herman GT (1970). Algebraic Reconstruction Techniques (ART) for three-dimensional electron microscopy and x-ray photography. J Theor Biol.

[31] Gordon R (1974). A tutorial on ART (Algebraic Reconstruction Techniques) [Erratum in Eq. 18: max, not min]. IEEE Trans Nucl Sci.

[32] Ma J (2010). Positively constrained multiplicative iterative algorithm for maximum penalized likelihood tomographic reconstruction. IEEE Trans Nucl Sci.

[33] Niu TY, Zhu L (2012). Accelerated barrier optimization compressed sensing (ABOCS) reconstruction for cone-beam CT: Phantom studies. Med Phys.

[34] Chen ZQ, Jin X, Li L, Wang G (2013). A limited-angle CT reconstruction method based on anisotropic TV minimization. Phys Med Biol.

[35] Niu S, Gao Y, Bian Z, Huang J, Chen W, Yu G (2014). Sparse-view x-ray CT reconstruction via total generalized variation regularization. Phys Med Biol.

[36] Zeng GSL, Gullberg GT (2013). On the bias of finite-view interior tomography using piecewise-constant and non-negativity constraints. Phys Med Biol.

[37] Mirone A, Brun E, Gouillart E, Tafforeau P, Kieffer J (2014). The PyHST2 hybrid distributed code for high speed tomographic reconstruction with iterative reconstruction and a priori knowledge capabilities. Nucl Instrum Methods Phys Res B: Beam Interactions with Materials and Atoms.

[38] Yoon S, Pineda AR, Fahrig R (2010). Simultaneous segmentation and reconstruction: A level set method approach for limited view computed tomography. Med Phys.

[39] Rangayyan RM, Gordon R (1982). Streak preventive image reconstruction with ART and adaptive filtering. IEEE Trans Med Imaging.

[40] Jin SO, Kim JG, Lee SY, Kwon OK (2012). Bone-induced streak artifact suppression in sparse-view CT image reconstruction. Biomed Eng Online.

[41] Y-z S, B-z W, Z-m Z (2008). Algebraic reconstruction techniques and improvement studied with spectroscopy. Spectrosc Spectr Anal.

[42] Ge Y, Li ZH, Wang ZX, He AZ, Lu AM (1996). Reconstruction of asymmetrical three-dimensional temperature field of radiator. Proc SPIE.

[43] Zhang B, He Y, Song Y, He AZ (2009). Deflection tomographic reconstruction of a complex flow field from incomplete projection data. Opt Lasers Eng.

[44] Mehta D, Thompson R, Morton T, Dhanantwari A, Shefer E (2013). Iterative model reconstruction: Simultaneously lowered computed tomography radiation dose and improved image quality. Med Phys Int J.

[45] Yasaka K, Katsura M, Akahane M, Sato J, Matsuda I, Ohtomo K (2014). Dose-reduced CT with model-based iterative reconstruction in evaluations of hepatic steatosis: How low can we go?. Eur J Radiol.

[46] Smith EA, Dillman JR, Goodsitt MM, Christodoulou EG, Keshavarzi N, Strouse PJ (2014). Model-based iterative reconstruction: Effect on patient radiation dose and image quality in pediatric body CT. Radiology.

[47] Schultze B, Witt M, Censor Y, Schulte R, Schubert KE. Performance of hull-detection algorithms for proton computed tomography reconstruction. In Contemporary Mathematics, Proceedings of the Workshop on Infinite Products of Operators and Their Applications, Technion, Haifa, Israel, May 21–24, 2012. 2014. [http://arxiv.org/abs/1402.1720]

[48] Kalos MH, Davis SA, Mittelman PS, Mastras P (1961). Conceptual Design of a Vapor Fraction Instrument.

[49] Mettler FA, Bhargavan M, Faulkner K, Gilley DB, Gray JE, Ibbott GS (2009). Radiologic and nuclear medicine studies in the United States and worldwide: Frequency, radiation dose, and comparison with other radiation sources—1950–2007. Radiology.

[50] Gordon R (1976). Dose reduction in computerized tomography [Guest Editorial]. Invest Radiol.

[51] Hara AK, Wellnitz CV, Paden RG, Pavlicek W, Sahani DV (2013). Reducing body CT radiation dose: beyond just changing the numbers. AJR Am J Roentgenol.

[52] Tekath M, Dutheil F, Bellini R, Roche A, Pereira B, Naughton G (2014). Comparison of the ultra-low-dose Veo algorithm with the gold standard filtered back projection for detecting pulmonary asbestos-related conditions: a clinical observational study. BMJ Open.

[53] Vardhanabhuti V, Riordan RD, Mitchell GR, Hyde C, Roobottom CA (2014). Image comparative assessment using iterative reconstructions clinical comparison of low-dose abdominal/pelvic computed tomography between adaptive statistical, model-based iterative reconstructions and traditional filtered back projection in 65 patients. Invest Radiol.

[54] Vinh-Hung V, Gordon R (2005). Quantitative target sizes for breast tumor detection prior to metastasis: a prerequisite to rational design of 4D scanners for breast screening. Technol Cancer Res Treat.

[55] Coumans FAW, Siesling S, Terstappen LWMM (2013). Detection of cancer before distant metastasis. BMC Cancer.

[56] McCollough CH, Yu L, Kofler JM, Leng S, Zhang Y, Li Z, et al. Degradation of CT low-contrast spatial resolution due to the use of iterative reconstruction and reduced dose levels. Radiology. 2015. (ahead of print): doi:10.1148/radiol.15142047. 10.1148/radiol.15142047 PMC4514568 25811326

[57] Apple Computer (1983). Macintosh MacPaint.

[58] Price GJ, Brunton AN, Beijersbergen MW, Fraser GW, Bavdaz M, Boutot JP (2002). X-ray focusing with Wolter microchannel plate optics. Nucl Instrum Methods Phys Res Section A-Accelerators Spectrometers Detectors and Associated Equipment.

[59] Vainshtein BK (1971). The synthesis of projecting functions. Sov Physics Dokl.

[60] Cappa P, Clerico A, Nov O, Porfiri M (2013). Can force feedback and science learning enhance the effectiveness of neuro-rehabilitation? An experimental study on using a low-cost 3D joystick and a virtual visit to a zoo. PLoS One.

[61] Bellman SH, Bender R, Gordon R, Rowe JE (1971). ART is science, being a defense of Algebraic Reconstruction Techniques for three-dimensional electron microscopy. J Theor Biol.

[62] Gordon R, Rangayyan RM (1983). Geometric deconvolution: a meta-algorithm for limited view computed tomography. IEEE Trans Biomed Eng.

[63] Dhawan AP, Rangayyan RM, Gordon R (1984). Wiener filtering for deconvolution of geometric artifacts in limited-view image reconstruction. Proc SPIE.

[64] Bamler R (1985). Comments on "Geometric deconvolution: A meta-algorithm for limited view computed-tomography". IEEE Trans Biomed Eng.

[65] Dhawan AP, Rangayyan RM, Gordon R (1985). Image restoration by Wiener deconvolution in limited-view computed tomography. Appl Optics.

[66] Gordon R, Dhawan AP, Rangayyan RM (1985). Reply to "Comments on geometric deconvolution: a meta-algorithm for limited view computed tomography". IEEE Trans Biomed Eng.

[67] Rangayyan RM, Dhawan AP, Gordon R (1985). Algorithms for limited-view computed tomography: an annotated bibliography and a challenge. Appl Optics.

[68] Antolak AJ, Lucadamo GA (2004). Nanoscale TEM tomography of metal oxide photocatalyst systems. Proc SPIE.

[69] Yu LF, Pan XC, Pelizzari CA, Martel M (2004). Few-view and limited-angle cone-beam megavoltage CT for breast localization in radiation therapy. Proc SPIE.

[70] Wan X, Zhang F, Chu Q, Zhang K, Sun F, Yuan B (2011). Three-dimensional reconstruction using an adaptive simultaneous algebraic reconstruction technique in electron tomography. J Struct Biol.

[71] Kisner SJ, Haneda E, Bouman CA, Skatter S, Kourinny M, Bedford S (2012). Limited view angle iterative CT reconstruction. Proc SPIE.

[72] Van de Sompel D, Brady M (2012). Regularising limited view tomography using anatomical reference images and information theoretic similarity metrics. Med Image Anal.

[73] Gordon R, Fu KS (1973). Artifacts in reconstructions made from a few projections. Proceedings of the First International Joint Conference on Pattern Recognition, Oct 30 to Nov 1, 1973, Washington, D C.

[74] Wikipedia. Internet protocol suite. 2014. [http://en.wikipedia.org/wiki/TCP/IP].

[75] Stein JA, Swift RD (1972). Flying spot x-ray imaging systems. Mater Eval.

[76] Beard DV, Pisano ED, Denelsbeck KM, Johnston RE (1994). Eye movement during computed tomography interpretation: eyetracker results and image display-time implications. J Digit Imaging.

[77] Tall M, Choudhury KR, Napel S, Roos JE, Rubin GD (2012). Accuracy of a remote eye tracker for radiologic observer studies: Effects of calibration and recording environment. Acad Radiol.

[78] Drew T, Vo ML, Olwal A, Jacobson F, Seltzer SE, Wolfe JM (2013). Scanners and drillers: Characterizing expert visual search through volumetric images. J Vis.

[79] Corcoran PM, Nanu F, Petrescu S, Bigioi P (2012). Real-time eye gaze tracking for gaming design and consumer electronics systems. IEEE Trans Consum Electron.

[80] Duchowski AT (2002). A breadth-first survey of eye-tracking applications. Behav Res Methods Instrum Comput.

[81] Kanade T, Hebert M (2012). First-person vision. Proc IEEE.

[82] Beard DV, Bream P, Pisano ED, Conroy P, Johnston RE, Braeuning P (1997). A pilot study of eye movement during mammography interpretation: eyetracker results and workstation design implications. J Digit Imaging.

[83] Matsumoto H, Terao Y, Yugeta A, Fukuda H, Emoto M, Furubayashi T (2011). Where do neurologists look when viewing brain CT images? An eye-tracking study involving stroke cases. PLoS One.

[84] Neault M. Tracking the Gaze. 2013. [http://blog.art21.org/2013/01/07/tracking-the-gaze/#.VHv4y4d4WZg]

[85] Katti H, Yadati K, Kankanhalli M, Tat-Seng C (2011). Affective video summarization and story board generation using pupillary dilation and eye gaze. 2011 IEEE International Symposium on Multimedia (ISM), 5-7 Dec 2011.

[86] Jain E, Sheikh Y, Hodgins J (2012). Inferring artistic intention in comic art through viewer gaze. Proceedings of the ACM Symposium on Applied Perception.

[87] Colquhoun GD, Gordon R, Tot T (2005). A superresolution computed tomography algorithm for reverse cone beam 3D x-ray mammography [PowerPoint presentation]. Workshop on Alternatives to Mammography, Copenhagen, September 29–30, 2005.

[88] Mishra D, Muralidhar K, Munshi P (1999). A robust MART algorithm for tomographic applications. Numerical Heat Transfer Part B-Fundamentals.

[89] Donaire JG, García I (2002). On using global optimization to obtain a better performance of a MART algorithm in 3D x-ray tomography. J Imaging Sci Technol.

[90] Badea C, Gordon R (2004). Experiments with the nonlinear and chaotic behaviour of the multiplicative algebraic reconstruction technique (MART) algorithm for computed tomography. Phys Med Biol.

[91] Bajpai M, Gupta P, Munshi P, Titarenko V, Withers PJ (2013). A graphical processing unit-based parallel implementation of Multiplicative Algebraic Reconstruction Technique algorithm for limited view tomography. Res Nondestruct Eval.

[92] Lakshminarayanan AV, Lent A (1979). Methods of least squares and SIRT in reconstruction. J Theor Biol.

[93] Gregor J, Benson T (2008). Computational analysis and improvement of SIRT. IEEE Trans Med Imaging.

[94] Gilbert P (1972). Iterative methods for the three-dimensional reconstruction of an object from projections. J Theor Biol.

[95] Pang WM, Qin J, Lu YQ, Xie YM, Chui CK, Heng PA (2011). Accelerating simultaneous algebraic reconstruction technique with motion compensation using CUDA-enabled GPU. Int J Comput Assist Radiol Surg.

[96] Xin JJ, Bardel C, Udpa L, Udpa S (2013). GPU implementation of simultaneous iterative reconstruction techniques for computed tomograpy. AIP Conference Proceedings.

[97] Kalarat K, Narkbuakaew W, Pintavirooj C, Sangworasil M (2006). Rapid simultaneous algebraic reconstruction technique (SART) for cone-beam geometry on clustering system. Proceedings TENCON 2005–2005 IEEE Region 10 Conference, Melbourne, Australia, November 21–24, 2005.

[98] Trummer MR (1981). Reconstructing pictures from projections: on the convergence of the ART algorithm with relaxation. Computing.

[99] Mazur EJ, Gordon R (1995). Interpolative algebraic reconstruction techniques without beam partitioning for computed tomography. Med Biol Eng Comput.

[100] Chakchouk M, Sevestre-Ghalila S, Graffigne C (2007). The benefit of a kernel estimate based forward projection for iterative tomogranphic reconstruction techniques. Proceedings of 29th Annual International Conference of the IEEE Engineering in Medicine and Biology Society, Cité Internationale, Lyon, France, August 23–26, 2007.

[101] Watt DW (1994). Column relaxed algebraic reconstruction algorithm for tomography with noisy data. Appl Optics.

[102] García I, Roca J, Sanjurjo J, Carazo JM, Zapata EL (1996). Implementation and experimental evaluation of the constrained ART algorithm on a multicomputer system. Signal Process.

[103] Melvin C, Thulasiraman P, Gordon R, Arabnia HR, Mun Y (2003). Parallel algebraic reconstruction technique for computed tomography. PDPTA'03: Proceedings of the International Conference on Parallel and Distributed Processing Techniques and Applications.

[104] Sourbelle K, Lauritsch G, Tam KC, Noo F, Kalender WA (2001). Performance evaluation of local ROI algorithms for exact ROI reconstruction in spiral cone-beam computed tomography. IEEE Trans Nucl Sci.

[105] Fager RS, Peddanarappagari KV, Kumar GN (1993). Pixel-based reconstruction (PBR) promising simultaneous techniques for CT reconstructions. IEEE Trans Med Imaging.

[106] Gordon R, Herman GT (1974). Three dimensional reconstruction from projections: a review of algorithms. Int Rev Cytol.

[107] Apfelmus H. Writing a paint program à la MS Paint - how to interpolate between mouse move events? 2010. [http://stackoverflow.com/questions/3347483/writing-a-paint-program-%C3%A0-la-ms-paint-how-to-interpolate-between-mouse-move-ev]

[108] Pawlak B, Gordon R (2005). Density estimation for positron emission tomography. Technol Cancer Res Treat.

[109] Gordon R, Menon D, Filipow LJ (1982). The ARTIST algorithm for high resolution, low dose positron tomography. Positron Emission Tomography, MARIA Design Symposium.

[110] Gordon R, Gordon R (1975). Maximal use of single photons and particles in reconstruction from projections by ARTIST, Algebraic Reconstruction Techniques Intended for Storage Tubes. Technical Digest, Topical Meeting on Image Processing for 2-D and 3-D Reconstruction from Projections: Theory and Practice in Medicine and the Physical Sciences.

[111] Sitek A (2008). Representation of photon limited data in emission tomography using origin ensembles. Phys Med Biol.

[112] Harmany ZT, Marcia RF, Willett RM (2010). Sparsity-regularized photon-limited imaging. 2010 7th IEEE International Symposium on Biomedical Imaging: From Nano to Macro.

[113] Harmany ZT, Marcia RF, Willett RM (2010). SPIRAL out of convexity: Sparsity-regularized algorithms for photon-limited imaging. Proc SPIE.

[114] Willett RM, Harmany ZT, Marcia RF (2010). Poisson image reconstruction with total variation regularization. 2010 IEEE International Conference on Image Processing.

[115] Sitek A, Moore SC (2013). Evaluation of imaging systems using the posterior variance of emission counts. IEEE Trans Med Imaging.

[116] Angel Luengo-Oroz M, Arranz A, Frean J (2012). Crowdsourcing malaria parasite quantification: An online game for analyzing images of infected thick blood smears. J Med Internet Res.

[117] Eiben CB, Siegel JB, Bale JB, Cooper S, Khatib F, Shen BW (2012). Increased Diels-Alderase activity through backbone remodeling guided by Foldit players. Nat Biotechnol.

[118] Schmidt M, Radchuk O, Meinhart C (2014). A serious game for public engagement in synthetic biology. Lect Notes Comput Sci.

[119] Good BM, Su AI (2011). Games with a scientific purpose. Genome Biol.

[120] Good BM, Su AI (2013). Crowdsourcing for bioinformatics. Bioinformatics.

[121] Rotman D, Preece J, Hammock J, Procita K, Hansen D, Parr C (2012). Dynamic changes in motivation in collaborative citizen-science projects. Proceedings of the ACM 2012 Conference on Computer Supported Cooperative Work.

[122] Lakhani KR, Boudreau KJ, Loh P-R, Backstrom L, Baldwin C, Lonstein E (2013). Prize-based contests can provide solutions to computational biology problems. Nat Biotechnol.

[123] Star K (2014). Doing useful work using games. Lect Notes Comput Sci.

[124] Curtis V (2014). Public engagement through the development of science-based computer games: The Wellcome Trust's "Gamify your PhD" initiative. Sci Commun.

[125] Schrope M (2013). Solving tough problems with games. Online communities are using the power of play to solve complex research problems. Proc Natl Acad Sci U S A.

[126] Waldispühl J, Kam A, Gardner P (2015). Crowdsourcing RNA structural alignments with an online computer game. Biocomputing 2015: Proceedings of the Pacific Symposium, Kohala Coast, Hawaii, USA, 4 – 8 January 2015.

[127] von Ahn L, Dabbish L (2004). Labeling images with a computer game. Proceedings of the SIGCHI Conference on Human factors in Computing Systems.

[128] von Ahn L, Liu R, Blum M (2006). Peekaboom: a game for locating objects in images. Proceedings of the SIGCHI Conference on Human Factors in Computing Systems.

[129] Alvare G, Gordon R. CT Brush game. 2014. [http://home.cc.umanitoba.ca/~alvare/ctbrush/index.html]

[130] Pekar V, Bystrov D, Heese HS, Dries SP, Schmidt S, Grewer R (2007). Automated planning of scan geometries in spine MRI scans. Medical Image Computing and Computer-Assisted Intervention–MICCAI 2007.

[131] Gordon R, Hirsch HVB, Schallenberger H, Schrey H (1977). Vision begins with direct reconstruction of the retinal image, how the brain sees and stores pictures. Gegenstrom, Für Helmut Hirsch zum Siebzigsten/Against the Stream, for Helmut Hirsch on His 70th Birthday.

[132] Gordon R, Tweed DB (1983). Quantitative reconstruction of visual cortex receptive fields. Univ Manitoba Med J.

[133] Guan H, Gordon R (1994). A projection access order for speedy convergence of ART (Algebraic Reconstruction Technique): a multilevel scheme for computed tomography. Phys Med Biol.

[134] Russ JC (2002). The Image Processing Handbook.

[135] Li H, Chen X, Wang Y, Zhou Z, Zhu Q, Yu D (2014). Sparse CT reconstruction based on multi-direction anisotropic total variation (MDATV). Biomed Eng Online.

[136] Cornely PRJ (2003). Flexible prior models: three-dimensional ionospheric tomography. Radio Sci.

[137] Battle XL, Cunningham GS, Hanson KM (1998). Tomographic reconstruction using 3D deformable models. Phys Med Biol.

[138] More MJ, Li H, Goodale PJ, Zheng YB, Majewski S, Popov V (2007). Limited angle dual modality breast imaging. IEEE Trans Nucl Sci.

[139] Erhard K, Grass M, Hitziger S, Iske A, Nielsen T (2012). Generalized filtered back-projection for digital breast tomosynthesis reconstruction. Proc SPIE.

[140] Qian X, Rajaram R, Calderon-Colon X, Yang G, Phan T, Lalush DS (2009). Design and characterization of a spatially distributed multibeam field emission x-ray source for stationary digital breast tomosynthesis. Med Phys.

[141] Bender R, Bellman SH, Gordon R (1970). ART and the ribosome: a preliminary report on the three-dimensional structure of individual ribosomes determined by an Algebraic Reconstruction Technique. J Theor Biol.

[142] Venkatakrishnan SV, Drummy LF, Jackson MA, De Graef M, Simmons J, Bouman CA (2013). A model based iterative reconstruction algorithm for High Angle Annular Dark Field-Scanning Transmission Electron Microscope (HAADF-STEM) Tomography. IEEE Trans Image Process.

[143] Fridman K, Mader A, Zwerger M, Elia N, Medalia O (2012). Advances in tomography: probing the molecular architecture of cells. Nat Rev Mol Cell Biol.

[144] Soble P, Rangayyan RM, Gordon R (1985). Quantitative and qualitative evaluation of geometric deconvolution of distortion in limited-view computed tomography. IEEE Trans Biomed Eng.

[145] Elangovan P, Warren LM, Mackenzie A, Rashidnasab A, Diaz O, Dance DR (2014). Development and validation of a modelling framework for simulating 2D-mammography and breast tomosynthesis images. Phys Med Biol.

[146] Moselk ApS. High performance software for large-scale LP, QP, SOCP, SDP and MIP including interfaces to C, Java, MATLAB, NET, R and Python. 2014. [https://mosek.com/]

[147] Jørgensen JS, Sidky EY. How little data is enough? Phase-diagram analysis of sparsity-regularized X-ray CT. 2014. [http://arxiv.org/abs/1412.6833] 10.1098/rsta.2014.0387 PMC4424483 25939620

[148] Rubinstein R, Zibulevsky M, Elad M (2010). Double sparsity: learning sparse dictionaries for sparse signal approximation. IEEE Trans Signal Process.

[149] Zhu L, Niu T, Petrongolo M (2014). Iterative CT reconstruction via minimizing adaptively reweighted total variation. J Xray Sci Technol.

[150] Yarbus AL (1967). Eye Movements and Vision.

[151] Starosta B. Help: How to Free-View the Stereo Pairs. 1999. [http://www.starosta.com/3dshowcase/ihelp.html]

[152] Shkolnik M. Mad Martian. 2014. [http://www.madmartian.com/shop/3-eyeballs]

