# Supplementary material for: CT brush and CancerZap!: two video games for computed tomography dose minimization
Source: Theor Biol Med Model. 2015 May 12;12:7. doi: 10.1186/s12976-015-0003-4 (PMC4469010; doi:10.1186/s12976-015-0003-4)
Supplement: Additional file 3: — The file ctdocs.zip is a zip file that contains all of the JavaDoc API documentation for the CT Brush project. All of the JavaDoc API documentation is in HTML format. To view this documentation, please load index.html (contained within this file) into a web-browser. [file 12976_2015_3_MOESM3_ESM.zip › docs/org/alvaregordon/ctbrush/GFXMath.html]

GFXMath


JavaScript is disabled on your browser.


- Package
- Class
- Use
- Tree
- Deprecated
- Index
- Help

*CT brush applet*

- Prev Class
- Next Class

- Frames
- No Frames

- All Classes

- Summary:
- Nested |
- Field |
- Constr |
- Method

- Detail:
- Field |
- Constr |
- Method


org.alvaregordon.ctbrush

## Class GFXMath

- java.lang.Object
- - org.alvaregordon.ctbrush.GFXMath

- ---

    

  ```
  public final class GFXMath
  extends java.lang.Object
  ```

  SYNOPSIS
  :   This class contains all of the mathematical algorithms for
      manipulating the hidden canvas (used by Main.genMap to generate
      the hidden canvas for each level), and the MART algorithm for
      brushing the workspace canvas. This class also contains the slope
      values for all of the possible lines used for MART projections
      (i.e. 0 to 179 degrees, see SLOPES).

  LICENSE
  :   This code is licensed under the Creative Commons 3.0

  Author:
  :   Graham Alvare, Richard Gordon

- - ### Field Summary

    Fields

    | Modifier and Type | Field and Description |
    | `static float[]` | `SLOPES` The tangent and co-tangents for every degree angles possible within the brush. |
  - ### Constructor Summary

    Constructors

    | Constructor and Description |
    | `GFXMath()` |
  - ### Method Summary

    Methods

    | Modifier and Type | Method and Description |
    | `static boolean` | `circleBresenhamFloat(int[] map, int mx, int my, int radius, int colour, int awidth, boolean check)` Bresenhanm Circle algorithm adapted from: http://www.sunshine2k.de/coding/java/Bresenham/RasterisingLinesCircles.pdf http://stackoverflow.com/questions/1201200/fast-algorithm-for-drawing-filled-circles http://en.wikipedia.org/wiki/Midpoint\_circle\_algorithm |
    | `static float` | `do_projection(int hash, int width, int height, float[] work, int[] hidden)` This function executes the MART algorithm using a given projection. |
    | `static boolean` | `drawLine(int[] map, int x1, int y1, int x2, int y2, int colour, int awidth, boolean check)` Digital Differential Analyzer line algorithm adapted from: http://www.sunshine2k.de/coding/java/Bresenham/RasterisingLinesCircles.pdf |
    | `static boolean` | `fillBottomFlatTriangle(int[] map, int x1, int y1, int x2, int y2, int x3, int y3, int colour, int awidth, boolean check)` Draws a triangle, which is flat at the bottom. |
    | `static boolean` | `fillTopFlatTriangle(int[] map, int x1, int y1, int x2, int y2, int x3, int y3, int colour, int awidth, boolean check)` Draws a triangle, which is flat at the top. |

    - ### Methods inherited from class java.lang.Object

      `clone, equals, finalize, getClass, hashCode, notify, notifyAll, toString, wait, wait, wait`

- - ### Field Detail


    - #### SLOPES

      ```
      public static float[] SLOPES
      ```

      The tangent and co-tangents for every degree angles possible within
      the brush. These are angles 0 to 179 degrees. Each index represents
      an angle (i.e. index 0 represents 0 degrees, index 179 represents 179
      degrees). The angles 0 to 179 were chosen, because (1) any line with
      an angular slope greater than 179 degrees is equal to a line with the
      same intercept value, but an angular slope that is between 0 and 179
      degrees; (2) the same is true for slopes less than 0 degrees.

      For reference, the angle is measured with the X-axis being zero
      degrees, and the Y-axis being 90 degrees.

      Note, the slope values are split between two functions, based on the
      angle they represent:

      1. **y = mx + b**: (the standard Y-axis line function, with the
         slope value m, and a Y-intercept of
         b).
      2. **x = ny + c**: (An X-axis line function, with a slope value
         of n), and an X-intercept value
         c.

      The Y-axis line function is used for angles in the ranges: [0,44] and
      [135,179] -- i.e. zero to 45 degrees, inclusive; and 135 degrees to
      179 degrees, inclusive.

      In contrast, the X-axis line function is used for angles between 45
      degrees and 134 degrees, inclusive.

      The slope values were split between two functions, so as to avoid the
      use of slope values greater than one. This is important, as it makes
      it easier to implement the MART iteration algorithm.
  - ### Constructor Detail


    - #### GFXMath

      ```
      public GFXMath()
      ```
  - ### Method Detail


    - #### fillTopFlatTriangle

      ```
      public static boolean fillTopFlatTriangle(int[] map,
                                int x1,
                                int y1,
                                int x2,
                                int y2,
                                int x3,
                                int y3,
                                int colour,
                                int awidth,
                                boolean check)
      ```

      Draws a triangle, which is flat at the top. This means that the
      first and second triangle vertices have the same y component.

      This method draws the triangle to a flattened 2D bitmap array
      (i.e. a 1D array which is indexed as if it were a 2D array).

      To minimize code duplication, this method also has a check feature.
      When the check feature is enabled, this method does not actually draw
      anything; instead, this method iterates through all of the pixels which
      correspond to the shape, and returns true if the value for each pixel is
      zero. Thus, this method may be used before drawing, to prevent image
      overlapping (i.e. drawing a triangle on-top of a circle).

      Developed based on code from:
      http://www.sunshine2k.de/coding/java/TriangleRasterization/TriangleRasterization.html#pointintrianglearticle

      Parameters:
      :   `map` - the map object to draw the triangle on. This array is
          indexed, such that each index corresponds to an X,Y
          coordinate-based point, where
          **index = X + Y \* awidth**.
      :   `x1` - the X-coordinate of the first vertex of the triangle.
      :   `y1` - the Y-coordinate of the first vertex of the triangle.
      :   `x2` - the X-coordinate of the second vertex of the triangle.
      :   `y2` - the Y-coordinate of the second vertex of the triangle.
      :   `x3` - the X-coordinate of the third vertex of the triangle.
      :   `y3` - the Y-coordinate of the third vertex of the triangle.
      :   `colour` - the numerical colour/grayscale code to write to the integer
          array matrix.
      :   `check` - if true, do NOT write to the map, but instead output
          whether the current shape will overwrite any existing data,
          if written, within the map.
      :   `awidth` - the width of each row in the map array. This is used for
          indexing a 2D array within a 1D array.

      Returns:
      :   true if either the check passed (i.e. data will not overlap), or
          the check flag is set to false (disabled). Otherwise, false.


    - #### fillBottomFlatTriangle

      ```
      public static boolean fillBottomFlatTriangle(int[] map,
                                   int x1,
                                   int y1,
                                   int x2,
                                   int y2,
                                   int x3,
                                   int y3,
                                   int colour,
                                   int awidth,
                                   boolean check)
      ```

      Draws a triangle, which is flat at the bottom. This means that the
      second and third triangle vertices have the same y component.

      This method draws the triangle to a flattened 2D bitmap array
      (i.e. a 1D array which is indexed as if it were a 2D array).

      To minimize code duplication, this method also has a check feature.
      When the check feature is enabled, this method does not actually draw
      anything; instead, this method iterates through all of the pixels which
      correspond to the shape, and returns true if the value for each pixel is
      zero. Thus, this method may be used before drawing, to prevent image
      overlapping (i.e. drawing a triangle on-top of a circle).

      Developed based on code from:
      http://www.sunshine2k.de/coding/java/TriangleRasterization/TriangleRasterization.html#pointintrianglearticle

      Parameters:
      :   `map` - the map object to draw the triangle on. This array is
          indexed, such that each index corresponds to an X,Y
          coordinate-based point, where
          **index = X + Y \* awidth**.
      :   `x1` - the X-coordinate of the first vertex of the triangle.
      :   `y1` - the Y-coordinate of the first vertex of the triangle.
      :   `x2` - the X-coordinate of the second vertex of the triangle.
      :   `y2` - the Y-coordinate of the second vertex of the triangle.
      :   `x3` - the X-coordinate of the third vertex of the triangle.
      :   `y3` - the Y-coordinate of the third vertex of the triangle.
      :   `colour` - the numerical colour/grayscale code to write to the integer
          array matrix.
      :   `check` - if true, do NOT write to the map, but instead output
          whether the current shape will overwrite any existing data,
          if written, within the map.
      :   `awidth` - the width of each row in the map array. This is used for
          indexing a 2D array within a 1D array.

      Returns:
      :   true if either the check passed (i.e. data will not overlap), or
          the check flag is set to false (disabled). Otherwise, false.


    - #### circleBresenhamFloat

      ```
      public static boolean circleBresenhamFloat(int[] map,
                                 int mx,
                                 int my,
                                 int radius,
                                 int colour,
                                 int awidth,
                                 boolean check)
      ```

      Bresenhanm Circle algorithm adapted from:
      http://www.sunshine2k.de/coding/java/Bresenham/RasterisingLinesCircles.pdf
      http://stackoverflow.com/questions/1201200/fast-algorithm-for-drawing-filled-circles
      http://en.wikipedia.org/wiki/Midpoint\_circle\_algorithm

      This method draws the circle to a flattened 2D bitmap array
      (i.e. a 1D array which is indexed as if it were a 2D array).

      To minimize code duplication, this method also has a check feature.
      When the check feature is enabled, this method does not actually draw
      anything; instead, this method iterates through all of the pixels which
      correspond to the shape, and returns true if the value for each pixel is
      zero. Thus, this method may be used before drawing, to prevent image
      overlapping (i.e. drawing a triangle on-top of a circle).

      Parameters:
      :   `map` - the map object to draw the circle on. This array is
          indexed, such that each index corresponds to an X,Y
          coordinate-based point, where
          **index = X + Y \* awidth**.
      :   `mx` - the X-coordinate of the centre of the circle.
      :   `my` - the Y-coordinate of the centre of the circle.
      :   `radius` - the radius of the circle.
      :   `colour` - the numerical colour/grayscale code to write to the integer
          array matrix.
      :   `check` - if true, do NOT write to the map, but instead output
          whether the current shape will overwrite any existing data,
          if written, within the map.
      :   `awidth` - the width of each row in the map array. This is used for
          indexing a 2D array within a 1D array.

      Returns:
      :   true if either the check passed (i.e. data will not overlap), or
          the check flag is set to false (disabled). Otherwise, false.


    - #### drawLine

      ```
      public static boolean drawLine(int[] map,
                     int x1,
                     int y1,
                     int x2,
                     int y2,
                     int colour,
                     int awidth,
                     boolean check)
      ```

      Digital Differential Analyzer line algorithm adapted from:
      http://www.sunshine2k.de/coding/java/Bresenham/RasterisingLinesCircles.pdf

      This method draws the line to a flattened 2D bitmap array
      (i.e. a 1D array which is indexed as if it were a 2D array).

      To minimize code duplication, this method also has a check feature.
      When the check feature is enabled, this method does not actually draw
      anything; instead, this method iterates through all of the pixels which
      correspond to the shape, and returns true if the value for each pixel is
      zero. Thus, this method may be used before drawing, to prevent image
      overlapping (i.e. drawing a triangle on-top of a circle).

      Parameters:
      :   `map` - the map object to draw the line on. This array is indexed,
          such that each index corresponds to an X,Y coordinate-based
          point, where **index = X + Y \* awidth**.
      :   `x1` - the X-coordinate of the first vertex of the line.
      :   `y1` - the Y-coordinate of the first vertex of the line.
      :   `x2` - the X-coordinate of the second vertex of the line.
      :   `y2` - the Y-coordinate of the second vertex of the line.
      :   `colour` - the numerical colour/grayscale code to write to the integer
          array matrix.
      :   `check` - if true, do NOT write to the map, but instead output
          whether the current shape will overwrite any existing data,
          if written, within the map.
      :   `awidth` - the width of each row in the map array. This is used for
          indexing a 2D array within a 1D array.

      Returns:
      :   true if either the check passed (i.e. data will not overlap), or
          the check flag is set to false (disabled). Otherwise, false.


    - #### do\_projection

      ```
      public static float do_projection(int hash,
                        int width,
                        int height,
                        float[] work,
                        int[] hidden)
      ```

      This function executes the MART algorithm using a given projection.
      MART stands for Multiplicative Arithmetic Reconstruction Technique.

      The projection is specified, using the following hash algorithm:

      ```
           angle + 180 * intercept
      ```

      Where angle refers to the angle around the X-axis (see SLOPES), and
      the intercept is the X or Y intercept of the appropriate line
      function to use for doing MART.

      Currently, the line for MART is traced using one of two functions:

      1. **y = mx + b**: (the standard Y-axis line function, with the
         slope value m, and a Y-intercept of
         b).
      2. **x = ny + c**: (An X-axis line function, with a slope value
         of n), and an X-intercept value
         c.

      Parameters:
      :   `hash` - the hash value of the projection to analyze.
      :   `width` - the width of the canvas to analyze.
      :   `height` - the height of the canvas to analyze.
      :   `work` - the array containing the MART estimation work. This array
          is indexed, such that each index corresponds to an X,Y
          coordinate-based point, where **index = X + Y \* width**.
      :   `hidden` - the array containing the hidden CT image. This array is
          indexed, such that each index corresponds to an X,Y
          coordinate-based point, where **index = X + Y \* width**.


- Package
- Class
- Use
- Tree
- Deprecated
- Index
- Help

*CT brush applet*

- Prev Class
- Next Class

- Frames
- No Frames

- All Classes

- Summary:
- Nested |
- Field |
- Constr |
- Method

- Detail:
- Field |
- Constr |
- Method

*Copyright © 2012 University of Manitoba.*
