# Supplementary material for: CT brush and CancerZap!: two video games for computed tomography dose minimization
Source: Theor Biol Med Model. 2015 May 12;12:7. doi: 10.1186/s12976-015-0003-4 (PMC4469010; doi:10.1186/s12976-015-0003-4)
Supplement: Additional file 3: — The file ctdocs.zip is a zip file that contains all of the JavaDoc API documentation for the CT Brush project. All of the JavaDoc API documentation is in HTML format. To view this documentation, please load index.html (contained within this file) into a web-browser. [file 12976_2015_3_MOESM3_ESM.zip › docs/org/alvaregordon/ctbrush/Main.html]

Main


JavaScript is disabled on your browser.


- Package
- Class
- Use
- Tree
- Deprecated
- Index
- Help

*CT brush applet*

- Prev Class
- Next Class

- Frames
- No Frames

- All Classes

- Summary:
- Nested |
- Field |
- Constr |
- Method

- Detail:
- Field |
- Constr |
- Method


org.alvaregordon.ctbrush

## Class Main

- java.lang.Object
- - java.awt.Component
  - - java.awt.Container
    - - java.awt.Panel
      - - java.applet.Applet
        - - javax.swing.JApplet
          - - org.alvaregordon.ctbrush.Main

- All Implemented Interfaces:
  :   java.awt.image.ImageObserver, java.awt.MenuContainer, java.io.Serializable, javax.accessibility.Accessible, javax.swing.RootPaneContainer

  ---

    

  ```
  public class Main
  extends javax.swing.JApplet
  ```

  SYNOPSIS
  :   A crow-source game applet designed to practice CT brush
      techniques.

  PARAMETERS
  :   Currently, the only parameters accepted by the applet are
      the track parameter and the port parameter. Each of these
      parameters is optional. If the track parameter is set to
      anything, other than blank, the CT brush applet will track
      the user's brush strokes and progress. By default, this
      tracking information will be sent to port 4444. However,
      the user may specify an alternate port, by passing a
      "port" parameter to the applet.

      The tracking information is represented as "pseudo-
      functions", where only one function is allowed on each
      line. Each level is preceded by a line containing ten (10)
      equal signs ("="). In addition, the triangles and
      circles describing a level are preceded, on each line, by four
      (4) space characters.

      Each pseudo-function is followed by parentheses. Inside the
      parentheses, parameters may be passed. These parameters are
      generally numbers, which are represented in the table below
      by the number sign ("#"); however, some of the pseudo-
      functions also pass boolean values (denoted as "bool")
      as parameters:

      |  |  |
      | --- | --- |
      | level(#:#:#) | indicates the user has progressed to a new level. The first number passed is the level number, the second number is the width of the level canvas, and the third is the height of the level canvas. |
      | t(#,#,#:bool) | indicates where a triangle is located within the current level canvas. The first 2 numbers are the x and y coordinates of the triangle. The next number is the size of the triangle. Because all of the triangles are right-angle isoceles triangles (with two 45 degree angles and one 90 degree angle), the size corresponds to either of the non- hypotenuse sides. Lastly, the boolean corresponds to whether the triangle is grey half-tone (true) or full tone (false). |
      | c(#,#,#:bool) | indicates where a circle is located within the current level canvas. The first 2 numbers are the x and y coordinates of the circle. The next number is the radius of the circle. Lastly, the boolean corresponds to whether the circle is a grey half- tone (true) or full tone (false). |
      | m(#,#:#^#\_#) | indicates a user mouse-brush movement within the level. The first two numbers, from the left, are the X and Y-coordinates, respectively; the third number corresponds to the width of the brush; the fourth number corresponds to the number of projections/rays/angles in the brush; the right- most number corresponds to the rotation of the brush. Currently, brush rotation is not implemented; however, it may be easily added in future versions. |
      | r() | indicates the user has chosen to perform a refinement action. |
      | g(#) | indicates the user has finished the level, and guessed the number of grey circles. The number passed by this pseudo-function is the user's guess. |

  LICENSE
  :   This code is licensed under the Creative Commons 3.0

  Author:
  :   Graham Alvare, Richard Gordon

  See Also:
  :   Serialized Form

- - ### Nested Class Summary

    - ### Nested classes/interfaces inherited from class javax.swing.JApplet

      `javax.swing.JApplet.AccessibleJApplet`
    - ### Nested classes/interfaces inherited from class java.applet.Applet

      `java.applet.Applet.AccessibleApplet`
    - ### Nested classes/interfaces inherited from class java.awt.Panel

      `java.awt.Panel.AccessibleAWTPanel`
    - ### Nested classes/interfaces inherited from class java.awt.Container

      `java.awt.Container.AccessibleAWTContainer`
    - ### Nested classes/interfaces inherited from class java.awt.Component

      `java.awt.Component.AccessibleAWTComponent, java.awt.Component.BaselineResizeBehavior, java.awt.Component.BltBufferStrategy, java.awt.Component.FlipBufferStrategy`
  - ### Field Summary

    Fields

    | Modifier and Type | Field and Description |
    | `javax.swing.JLabel` | `BRUSH_AREA` The JLabel to display the canvas to the user. |
    | `javax.swing.AbstractAction` | `REFINE_ACTION` A menu item for performing refinement iterations on the canvas data. |
    | `javax.swing.JApplet` | `SELF` A self-reference to the JApplet object. |

    - ### Fields inherited from class javax.swing.JApplet

      `accessibleContext, rootPane, rootPaneCheckingEnabled`
    - ### Fields inherited from class java.awt.Component

      `BOTTOM_ALIGNMENT, CENTER_ALIGNMENT, LEFT_ALIGNMENT, RIGHT_ALIGNMENT, TOP_ALIGNMENT`
    - ### Fields inherited from interface java.awt.image.ImageObserver

      `ABORT, ALLBITS, ERROR, FRAMEBITS, HEIGHT, PROPERTIES, SOMEBITS, WIDTH`
  - ### Constructor Summary

    Constructors

    | Constructor and Description |
    | `Main()` |
  - ### Method Summary

    Methods

    | Modifier and Type | Method and Description |
    | `java.awt.Point` | `getCanvasLocation()` |
    | `boolean` | `hasProjection(int alter, short angle)` Tests if a projection has already been "shot" (read) from the workspace. |
    | `void` | `init()` Initializes the CT brush Applet. |
    | `void` | `mouseBrush(int x0, int y0, byte wray, short nray, byte brota)` Performs a mouse CT-brush operation |
    | `void` | `updateImage(boolean showcursor)` Updates the current canvas image. |
    | `void` | `updateStatus()` |

    - ### Methods inherited from class javax.swing.JApplet

      `addImpl, createRootPane, getAccessibleContext, getContentPane, getGlassPane, getGraphics, getJMenuBar, getLayeredPane, getRootPane, getTransferHandler, isRootPaneCheckingEnabled, paramString, remove, repaint, setContentPane, setGlassPane, setJMenuBar, setLayeredPane, setLayout, setRootPane, setRootPaneCheckingEnabled, setTransferHandler, update`
    - ### Methods inherited from class java.applet.Applet

      `destroy, getAppletContext, getAppletInfo, getAudioClip, getAudioClip, getCodeBase, getDocumentBase, getImage, getImage, getLocale, getParameter, getParameterInfo, isActive, isValidateRoot, newAudioClip, play, play, resize, resize, setStub, showStatus, start, stop`
    - ### Methods inherited from class java.awt.Panel

      `addNotify`
    - ### Methods inherited from class java.awt.Container

      `add, add, add, add, add, addContainerListener, addPropertyChangeListener, addPropertyChangeListener, applyComponentOrientation, areFocusTraversalKeysSet, countComponents, deliverEvent, doLayout, findComponentAt, findComponentAt, getAlignmentX, getAlignmentY, getComponent, getComponentAt, getComponentAt, getComponentCount, getComponents, getComponentZOrder, getContainerListeners, getFocusTraversalKeys, getFocusTraversalPolicy, getInsets, getLayout, getListeners, getMaximumSize, getMinimumSize, getMousePosition, getPreferredSize, insets, invalidate, isAncestorOf, isFocusCycleRoot, isFocusCycleRoot, isFocusTraversalPolicyProvider, isFocusTraversalPolicySet, layout, list, list, locate, minimumSize, paint, paintComponents, preferredSize, print, printComponents, processContainerEvent, processEvent, remove, removeAll, removeContainerListener, removeNotify, setComponentZOrder, setFocusCycleRoot, setFocusTraversalKeys, setFocusTraversalPolicy, setFocusTraversalPolicyProvider, setFont, transferFocusDownCycle, validate, validateTree`
    - ### Methods inherited from class java.awt.Component

      `action, add, addComponentListener, addFocusListener, addHierarchyBoundsListener, addHierarchyListener, addInputMethodListener, addKeyListener, addMouseListener, addMouseMotionListener, addMouseWheelListener, bounds, checkImage, checkImage, coalesceEvents, contains, contains, createImage, createImage, createVolatileImage, createVolatileImage, disable, disableEvents, dispatchEvent, enable, enable, enableEvents, enableInputMethods, firePropertyChange, firePropertyChange, firePropertyChange, firePropertyChange, firePropertyChange, firePropertyChange, firePropertyChange, firePropertyChange, firePropertyChange, getBackground, getBaseline, getBaselineResizeBehavior, getBounds, getBounds, getColorModel, getComponentListeners, getComponentOrientation, getCursor, getDropTarget, getFocusCycleRootAncestor, getFocusListeners, getFocusTraversalKeysEnabled, getFont, getFontMetrics, getForeground, getGraphicsConfiguration, getHeight, getHierarchyBoundsListeners, getHierarchyListeners, getIgnoreRepaint, getInputContext, getInputMethodListeners, getInputMethodRequests, getKeyListeners, getLocation, getLocation, getLocationOnScreen, getMouseListeners, getMouseMotionListeners, getMousePosition, getMouseWheelListeners, getName, getParent, getPeer, getPropertyChangeListeners, getPropertyChangeListeners, getSize, getSize, getToolkit, getTreeLock, getWidth, getX, getY, gotFocus, handleEvent, hasFocus, hide, imageUpdate, inside, isBackgroundSet, isCursorSet, isDisplayable, isDoubleBuffered, isEnabled, isFocusable, isFocusOwner, isFocusTraversable, isFontSet, isForegroundSet, isLightweight, isMaximumSizeSet, isMinimumSizeSet, isOpaque, isPreferredSizeSet, isShowing, isValid, isVisible, keyDown, keyUp, list, list, list, location, lostFocus, mouseDown, mouseDrag, mouseEnter, mouseExit, mouseMove, mouseUp, move, nextFocus, paintAll, postEvent, prepareImage, prepareImage, printAll, processComponentEvent, processFocusEvent, processHierarchyBoundsEvent, processHierarchyEvent, processInputMethodEvent, processKeyEvent, processMouseEvent, processMouseMotionEvent, processMouseWheelEvent, remove, removeComponentListener, removeFocusListener, removeHierarchyBoundsListener, removeHierarchyListener, removeInputMethodListener, removeKeyListener, removeMouseListener, removeMouseMotionListener, removeMouseWheelListener, removePropertyChangeListener, removePropertyChangeListener, repaint, repaint, repaint, requestFocus, requestFocus, requestFocusInWindow, requestFocusInWindow, reshape, revalidate, setBackground, setBounds, setBounds, setComponentOrientation, setCursor, setDropTarget, setEnabled, setFocusable, setFocusTraversalKeysEnabled, setForeground, setIgnoreRepaint, setLocale, setLocation, setLocation, setMaximumSize, setMinimumSize, setName, setPreferredSize, setSize, setSize, setVisible, show, show, size, toString, transferFocus, transferFocusBackward, transferFocusUpCycle`
    - ### Methods inherited from class java.lang.Object

      `clone, equals, finalize, getClass, hashCode, notify, notifyAll, wait, wait, wait`

- - ### Field Detail


    - #### BRUSH\_AREA

      ```
      public final javax.swing.JLabel BRUSH_AREA
      ```

      The JLabel to display the canvas to the user.


    - #### SELF

      ```
      public final javax.swing.JApplet SELF
      ```

      A self-reference to the JApplet object. For use in inner-classes.


    - #### REFINE\_ACTION

      ```
      public final javax.swing.AbstractAction REFINE_ACTION
      ```

      A menu item for performing refinement iterations on the canvas data.
  - ### Constructor Detail


    - #### Main

      ```
      public Main()
      ```
  - ### Method Detail


    - #### init

      ```
      public void init()
      ```

      Initializes the CT brush Applet.
      This involves reading data from the server for the images
      to be displayed, reading and setting up the current hidden image,
      initializing the mouse tracker/handler, and preparing the program
      to calculate and analyze raysums from the user's brush projections.

      **Overrides:**
      :   `init` in class `java.applet.Applet`


    - #### updateStatus

      ```
      public void updateStatus()
      ```


    - #### updateImage

      ```
      public void updateImage(boolean showcursor)
      ```

      Updates the current canvas image.
      This method first refreshes the workspace canvas, then calls
      drawCursor to draw the brush cursor on top of the workspace canvas.

      Parameters:
      :   `showcursor` - if set to false, the mouse cursor will NOT be shown.


    - #### getCanvasLocation

      ```
      public java.awt.Point getCanvasLocation()
      ```


    - #### mouseBrush

      ```
      public void mouseBrush(int x0,
                    int y0,
                    byte wray,
                    short nray,
                    byte brota)
      ```

      Performs a mouse CT-brush operation

      How to BRUSH  
      Select the number of angles (N)  
      Select rotation angle for the brush (A0 < 180/N)

      This method performs Multiplicative Arithmetic Reconstruction
      Technique (MART) calculations on a projection. MART is used to estimate
      the pixels/voxels of resulting image from the list of projections.

      The MART algorithm used is as follows:
      :   `Point (x,y,time + 1) = Point(x,y,time + 1) * [ Raysum(time + 1) / Raysum(time) ]`

      *Projection line function: (y = mx + b)*

      This algorithm should skip zeros, because if Point(x,y,time + 1) = 0,
      then the point will stay zero for all future point estimations
      (anything multiplied by zero yields zero).

      Parameters:
      :   `x0` - the x position of the mouse cursor
      :   `y0` - the y position of the mouse cursor
      :   `wray` - - the width of the cursor brush
      :   `nray` - - the number of angles involves with the cursor brush
      :   `brota` - - the angle of rotation of the cursor brush


    - #### hasProjection

      ```
      public boolean hasProjection(int alter,
                          short angle)
      ```

      Tests if a projection has already been "shot" (read) from the workspace.

      Parameters:
      :   `alter` - the b/c value for the line equation.
      :   `angle` - the angle of the slope of the line (in degrees, clockwise;
          where 0 is the X-axis).


- Package
- Class
- Use
- Tree
- Deprecated
- Index
- Help

*CT brush applet*

- Prev Class
- Next Class

- Frames
- No Frames

- All Classes

- Summary:
- Nested |
- Field |
- Constr |
- Method

- Detail:
- Field |
- Constr |
- Method

*Copyright © 2012 University of Manitoba.*
