# Supplementary material for: CT brush and CancerZap!: two video games for computed tomography dose minimization
Source: Theor Biol Med Model. 2015 May 12;12:7. doi: 10.1186/s12976-015-0003-4 (PMC4469010; doi:10.1186/s12976-015-0003-4)
Supplement: Additional file 3: — The file ctdocs.zip is a zip file that contains all of the JavaDoc API documentation for the CT Brush project. All of the JavaDoc API documentation is in HTML format. To view this documentation, please load index.html (contained within this file) into a web-browser. [file 12976_2015_3_MOESM3_ESM.zip › docs/org/alvaregordon/ctbrush/MouseHandler.html]

MouseHandler


JavaScript is disabled on your browser.


- Package
- Class
- Use
- Tree
- Deprecated
- Index
- Help

*CT brush applet*

- Prev Class
- Next Class

- Frames
- No Frames

- All Classes

- Summary:
- Nested |
- Field |
- Constr |
- Method

- Detail:
- Field |
- Constr |
- Method


org.alvaregordon.ctbrush

## Class MouseHandler

- java.lang.Object
- - java.awt.event.MouseAdapter
  - - org.alvaregordon.ctbrush.MouseHandler

- All Implemented Interfaces:
  :   java.awt.event.MouseListener, java.awt.event.MouseMotionListener, java.awt.event.MouseWheelListener, java.util.EventListener

  ---

    

  ```
  public class MouseHandler
  extends java.awt.event.MouseAdapter
  ```

  SYNOPSIS
  :   The mouse handler. This object handles all mouse events for the
      program.

  LICENSE
  :   This code is licensed under the Creative Commons 3.0

  Author:
  :   Graham Alvare, Richard Gordon

- - ### Field Summary

    Fields

    | Modifier and Type | Field and Description |
    | `static short` | `NRAY_MAX` The maximum number of X-ray projections that can be used in the brush. |
    | `static byte` | `NRAY_MIN` The minimum number of X-ray projections that can be used in the brush. |
    | `javax.swing.AbstractAction` | `NRAY_MINUS` The increase number of rays button. |
    | `javax.swing.AbstractAction` | `NRAY_PLUS` The increase number of rays button. |
    | `static byte` | `NRAY_STEP` The increment step for number of X-ray projections to use in the brush. |
    | `static byte` | `WRAY_MAX` The maximum width of X-ray projections that can be used in the brush. |
    | `static byte` | `WRAY_MIN` The minimum width of X-ray projections that can be used in the brush. |
    | `javax.swing.AbstractAction` | `WRAY_MINUS` The decrease width of X-rays button. |
    | `javax.swing.AbstractAction` | `WRAY_PLUS` The increase width of X-rays button. |
    | `static byte` | `WRAY_STEP` The increment step for width of X-ray projections to use in the brush. |
  - ### Constructor Summary

    Constructors

    | Constructor and Description |
    | `MouseHandler(Main canvas)` This method creates a new mouse handler object for the CT brush canvas. |
  - ### Method Summary

    Methods

    | Modifier and Type | Method and Description |
    | `void` | `drawCursor(java.awt.Graphics gfx, int width, int height)` Draws the brush at the current cursor position, so the user can see what they are doing - they can see what area clicking the mouse should uncover. |
    | `int` | `getNrays()` |
    | `int` | `getWrays()` |
    | `void` | `mouseClicked(java.awt.event.MouseEvent e)` Performs a CT brush event when the mouse is clicked. |
    | `void` | `mouseDragged(java.awt.event.MouseEvent e)` Handles dragging the mouse. |
    | `void` | `mouseMoved(java.awt.event.MouseEvent e)` Draws the brush at the current cursor position, so the user can see what they are doing - they can see what area clicking the mouse should uncover. |
    | `void` | `mouseReleased(java.awt.event.MouseEvent e)` Clean up interpolation for dragging. |
    | `void` | `mouseWheelMoved(java.awt.event.MouseWheelEvent e)` Sense mouse wheel movements and increment or decrement the number of rays in the mouse cursor. |

    - ### Methods inherited from class java.awt.event.MouseAdapter

      `mouseEntered, mouseExited, mousePressed`
    - ### Methods inherited from class java.lang.Object

      `clone, equals, finalize, getClass, hashCode, notify, notifyAll, toString, wait, wait, wait`

- - ### Field Detail


    - #### NRAY\_MIN

      ```
      public static final byte NRAY_MIN
      ```

      The minimum number of X-ray projections that can be used in the brush.

      See Also:
      :   Constant Field Values


    - #### NRAY\_MAX

      ```
      public static final short NRAY_MAX
      ```

      The maximum number of X-ray projections that can be used in the brush.

      See Also:
      :   Constant Field Values


    - #### NRAY\_STEP

      ```
      public static final byte NRAY_STEP
      ```

      The increment step for number of X-ray projections to use in the brush.

      See Also:
      :   Constant Field Values


    - #### WRAY\_MIN

      ```
      public static final byte WRAY_MIN
      ```

      The minimum width of X-ray projections that can be used in the brush.

      See Also:
      :   Constant Field Values


    - #### WRAY\_MAX

      ```
      public static final byte WRAY_MAX
      ```

      The maximum width of X-ray projections that can be used in the brush.

      See Also:
      :   Constant Field Values


    - #### WRAY\_STEP

      ```
      public static final byte WRAY_STEP
      ```

      The increment step for width of X-ray projections to use in the brush.

      See Also:
      :   Constant Field Values


    - #### NRAY\_PLUS

      ```
      public final javax.swing.AbstractAction NRAY_PLUS
      ```

      The increase number of rays button. This button increases the number
      of X-rays used in the CT brush.


    - #### NRAY\_MINUS

      ```
      public final javax.swing.AbstractAction NRAY_MINUS
      ```

      The increase number of rays button. This button increases the number
      of X-rays used in the CT brush.


    - #### WRAY\_PLUS

      ```
      public final javax.swing.AbstractAction WRAY_PLUS
      ```

      The increase width of X-rays button.
      This button increases the width of X-rays used in the CT brush.


    - #### WRAY\_MINUS

      ```
      public final javax.swing.AbstractAction WRAY_MINUS
      ```

      The decrease width of X-rays button.
      This button decreases the width of X-rays used in the CT brush.
  - ### Constructor Detail


    - #### MouseHandler

      ```
      public MouseHandler(Main canvas)
      ```

      This method creates a new mouse handler object for the CT brush canvas.

      Parameters:
      :   `canvas` - the parent canvas to associate the mouse handler to.
  - ### Method Detail


    - #### mouseDragged

      ```
      public void mouseDragged(java.awt.event.MouseEvent e)
      ```

      Handles dragging the mouse. This method handles mouse drag events by
      brushing the area traced by the mouse, with the CT X-ray projections.
      Because this method is not called for every pixel the user drags his or
      her mouse, gaps in the mouse drag are produced. Therefore, this method
      performs basic linear interpretation to minimize/correct these gaps.
      The linear interpolation algorithm used is the standard Bressenahm's
      line algorithm.

      NOTE: The mouse on OS X tracks at about 1 sample / 16.5 ms
      independently of the tracking speed of the mouse.

      **Specified by:**
      :   `mouseDragged` in interface `java.awt.event.MouseMotionListener`

      **Overrides:**
      :   `mouseDragged` in class `java.awt.event.MouseAdapter`

      Parameters:
      :   `e` - the mouse drag event to obtain cursor information from.


    - #### mouseMoved

      ```
      public void mouseMoved(java.awt.event.MouseEvent e)
      ```

      Draws the brush at the current cursor position, so the user can see what
      they are doing - they can see what area clicking the mouse should
      uncover.

      **Specified by:**
      :   `mouseMoved` in interface `java.awt.event.MouseMotionListener`

      **Overrides:**
      :   `mouseMoved` in class `java.awt.event.MouseAdapter`

      Parameters:
      :   `e` - used to obtain x and y coordinates


    - #### mouseClicked

      ```
      public void mouseClicked(java.awt.event.MouseEvent e)
      ```

      Performs a CT brush event when the mouse is clicked.
      Raysums are calculated, the projection is analyzed via MART,
      and the radiation dose to the patient is incremented.

      **Specified by:**
      :   `mouseClicked` in interface `java.awt.event.MouseListener`

      **Overrides:**
      :   `mouseClicked` in class `java.awt.event.MouseAdapter`

      Parameters:
      :   `e` - used to obtain x and y coordinates


    - #### mouseReleased

      ```
      public void mouseReleased(java.awt.event.MouseEvent e)
      ```

      Clean up interpolation for dragging.
      Because interpolation relies on tracing the mouse cursor from
      'lastpoint' to the current mouse position, 'lastpoint' MUST be
      reset to null (i.e. disabled) whenever the mouse button is
      released.

      **Specified by:**
      :   `mouseReleased` in interface `java.awt.event.MouseListener`

      **Overrides:**
      :   `mouseReleased` in class `java.awt.event.MouseAdapter`

      Parameters:
      :   `e` - the mouse event - this is ignored


    - #### mouseWheelMoved

      ```
      public void mouseWheelMoved(java.awt.event.MouseWheelEvent e)
      ```

      Sense mouse wheel movements and increment or decrement the number of rays
      in the mouse cursor. The number of rays is incremented when the mouse
      wheel is scrolled up, and decremented when the mouse wheel is scrolled
      down; the magnitude of the increase or decrease is based on the number
      of notches the mouse wheel was scrolled. Each notch scrolled is
      equivalent to one call to the actionPerformed method of either NRAY\_PLUS
      or NRAY\_MINUS.

      **Specified by:**
      :   `mouseWheelMoved` in interface `java.awt.event.MouseWheelListener`

      **Overrides:**
      :   `mouseWheelMoved` in class `java.awt.event.MouseAdapter`


    - #### getWrays

      ```
      public int getWrays()
      ```


    - #### getNrays

      ```
      public int getNrays()
      ```


    - #### drawCursor

      ```
      public void drawCursor(java.awt.Graphics gfx,
                    int width,
                    int height)
      ```

      Draws the brush at the current cursor position, so the user can
      see what they are doing - they can see what area clicking the mouse
      should uncover.
      NOTE: The mouse on OS X tracks at about 1 sample / 16.5 ms
      independently of the tracking speed of the mouse.
      Therefore, the mouse movements MUST be interpolated,
      when the mouse is dragged. Linear interpolation is used.

      Parameters:
      :   `gfx` - - the Graphics object to draw the cursor with
      :   `width` - - the width of the destination image
      :   `height` - - the height of the destination image


- Package
- Class
- Use
- Tree
- Deprecated
- Index
- Help

*CT brush applet*

- Prev Class
- Next Class

- Frames
- No Frames

- All Classes

- Summary:
- Nested |
- Field |
- Constr |
- Method

- Detail:
- Field |
- Constr |
- Method

*Copyright © 2012 University of Manitoba.*
