# Supplementary material for: CT brush and CancerZap!: two video games for computed tomography dose minimization
Source: Theor Biol Med Model. 2015 May 12;12:7. doi: 10.1186/s12976-015-0003-4 (PMC4469010; doi:10.1186/s12976-015-0003-4)
Supplement: Additional file 3: — The file ctdocs.zip is a zip file that contains all of the JavaDoc API documentation for the CT Brush project. All of the JavaDoc API documentation is in HTML format. To view this documentation, please load index.html (contained within this file) into a web-browser. [file 12976_2015_3_MOESM3_ESM.zip › docs/org/alvaregordon/ctbrush/Workspace.html]

Workspace


JavaScript is disabled on your browser.


- Package
- Class
- Use
- Tree
- Deprecated
- Index
- Help

*CT brush applet*

- Prev Class
- Next Class

- Frames
- No Frames

- All Classes

- Summary:
- Nested |
- Field |
- Constr |
- Method

- Detail:
- Field |
- Constr |
- Method


org.alvaregordon.ctbrush

## Class Workspace

- java.lang.Object
- - org.alvaregordon.ctbrush.Workspace

- All Implemented Interfaces:
  :   java.lang.Runnable

  ---

    

  ```
  public final class Workspace
  extends java.lang.Object
  implements java.lang.Runnable
  ```

  SYNOPSIS
  :   The workspace canvas for the CT brush. This canvas stores all of
      the data which describes the current level and the user's progress.
      In addition, this canvas contains methods for performing CT brush
      functions, such as adding projections and displaying the current
      status of the canvas. Also, this method implements the Runnable
      interface, so that MART computations can be executed in a separate
      thread from other CT brush operations.

  LICENSE
  :   This code is licensed under the Creative Commons 3.0

  Author:
  :   Graham Alvare, Richard Gordon

- - ### Constructor Summary

    Constructors

    | Constructor and Description |
    | `Workspace(Main canvas, int width, int height, int[] hidden)` Creates a new workspace from a 2D hidden image (represented as a 1D array object; indices are computed as [Y \* width + X]). |
  - ### Method Summary

    Methods

    | Modifier and Type | Method and Description |
    | `boolean` | `addProjection(int alter, int angle)` Adds a projection to the workspace. |
    | `void` | `displayImage(java.awt.image.BufferedImage canvasImage)` Display the current workspace image in the user's canvas. |
    | `protected void` | `finalize()` |
    | `double` | `getDose()` Calculates the total amount of dose administered for the current leve. |
    | `boolean` | `hasProjection(int alter, int angle)` Checks if a projection was already added to the workspace. |
    | `void` | `refine(java.awt.Frame parent)` Performs a refinement MART iteration on the canvas. |
    | `void` | `run()` Performs MART operations (based on the user's mouse clicks) in a separate thread. |
    | `void` | `showHidden(java.awt.image.BufferedImage canvasImage)` Display the hidden image for the workspace |

    - ### Methods inherited from class java.lang.Object

      `clone, equals, getClass, hashCode, notify, notifyAll, toString, wait, wait, wait`

- - ### Constructor Detail


    - #### Workspace

      ```
      public Workspace(Main canvas,
               int width,
               int height,
               int[] hidden)
      ```

      Creates a new workspace from a 2D hidden image (represented as a 1D
      array object; indices are computed as [Y \* width + X]).

      Parameters:
      :   `canvas` - the main canvas to use for updating the user's status.
      :   `width` - the width of the hidden image.
      :   `height` - the height of the hidden image.
      :   `hidden` - the hidden image.
  - ### Method Detail


    - #### getDose

      ```
      public final double getDose()
      ```

      Calculates the total amount of dose administered for the current leve.
      This value is equivalent to the user's current score (for the level).

      Returns:
      :   the total dose administerd for the workspace/level.


    - #### displayImage

      ```
      public void displayImage(java.awt.image.BufferedImage canvasImage)
      ```

      Display the current workspace image in the user's canvas.

      Parameters:
      :   `canvasImage` - the image object to output the current workspace data
          to. (The current workspace data is housed in the
          variable "work".)


    - #### showHidden

      ```
      public void showHidden(java.awt.image.BufferedImage canvasImage)
      ```

      Display the hidden image for the workspace

      Parameters:
      :   `canvasImage` - the image object to ouput the hidden image to.


    - #### addProjection

      ```
      public boolean addProjection(int alter,
                          int angle)
      ```

      Adds a projection to the workspace.

      Currently, the projection lines aretraced using one of two functions:

      1. **y = mx + b**: (the standard Y-axis line function, with the
         slope value m, and a Y-intercept of
         b).
      2. **x = ny + c**: (An X-axis line function, with a slope value
         of n), and an X-intercept value
         c.

      Parameters:
      :   `alter` - the alter (b or c) to the line equation for the projection.
      :   `angle` - the angle (in degrees) of the projection.

      Returns:
      :   true if the projection was added successfully (i.e. the
          projection was not previously added.


    - #### hasProjection

      ```
      public boolean hasProjection(int alter,
                          int angle)
      ```

      Checks if a projection was already added to the workspace.

      Currently, the projection lines aretraced using one of two functions:

      1. **y = mx + b**: (the standard Y-axis line function, with the
         slope value m, and a Y-intercept of
         b).
      2. **x = ny + c**: (An X-axis line function, with a slope value
         of n), and an X-intercept value
         c.

      Parameters:
      :   `alter` - the alter (b or c) to the line equation for the projection.
      :   `angle` - the angle (in degrees) of the projection.

      Returns:
      :   true if the projection was previously added to the workspace.


    - #### run

      ```
      public void run()
      ```

      Performs MART operations (based on the user's mouse clicks) in a
      separate thread. This is done to help keep the user interface
      quick and responsive.

      **Specified by:**
      :   `run` in interface `java.lang.Runnable`


    - #### refine

      ```
      public void refine(java.awt.Frame parent)
      ```

      Performs a refinement MART iteration on the canvas.

      Parameters:
      :   `parent` - the parent frame for creating a modal progress dialog box.


    - #### finalize

      ```
      protected void finalize()
      ```

      **Overrides:**
      :   `finalize` in class `java.lang.Object`


- Package
- Class
- Use
- Tree
- Deprecated
- Index
- Help

*CT brush applet*

- Prev Class
- Next Class

- Frames
- No Frames

- All Classes

- Summary:
- Nested |
- Field |
- Constr |
- Method

- Detail:
- Field |
- Constr |
- Method

*Copyright © 2012 University of Manitoba.*
