# Supplementary material for: CT brush and CancerZap!: two video games for computed tomography dose minimization
Source: Theor Biol Med Model. 2015 May 12;12:7. doi: 10.1186/s12976-015-0003-4 (PMC4469010; doi:10.1186/s12976-015-0003-4)
Supplement: Additional file 3: — The file ctdocs.zip is a zip file that contains all of the JavaDoc API documentation for the CT Brush project. All of the JavaDoc API documentation is in HTML format. To view this documentation, please load index.html (contained within this file) into a web-browser. [file 12976_2015_3_MOESM3_ESM.zip › docs/org/alvaregordon/ctbrush/package-summary.html]

org.alvaregordon.ctbrush


JavaScript is disabled on your browser.


- Package
- Class
- Use
- Tree
- Deprecated
- Index
- Help

*CT brush applet*

- Prev Package
- Next Package

- Frames
- No Frames

- All Classes

# Package org.alvaregordon.ctbrush

- Class Summary

  | Class | Description |
  |  |  |
  | --- | --- |
  | GFXMath | SYNOPSIS This class contains all of the mathematical algorithms for manipulating the hidden canvas (used by Main.genMap to generate the hidden canvas for each level), and the MART algorithm for brushing the workspace canvas. |
  | Main | SYNOPSIS |
  | MouseHandler | SYNOPSIS The mouse handler. |
  | Workspace | SYNOPSIS The workspace canvas for the CT brush. |

- Package
- Class
- Use
- Tree
- Deprecated
- Index
- Help

*CT brush applet*

- Prev Package
- Next Package

- Frames
- No Frames

- All Classes

*Copyright © 2012 University of Manitoba.*
