# Supplementary material for: CT brush and CancerZap!: two video games for computed tomography dose minimization
Source: Theor Biol Med Model. 2015 May 12;12:7. doi: 10.1186/s12976-015-0003-4 (PMC4469010; doi:10.1186/s12976-015-0003-4)
Supplement: Additional file 3: — The file ctdocs.zip is a zip file that contains all of the JavaDoc API documentation for the CT Brush project. All of the JavaDoc API documentation is in HTML format. To view this documentation, please load index.html (contained within this file) into a web-browser. [file 12976_2015_3_MOESM3_ESM.zip › docs/org/alvaregordon/ctbrush/class-use/Main.html]

Uses of Class org.alvaregordon.ctbrush.Main


JavaScript is disabled on your browser.


- Package
- Class
- Use
- Tree
- Deprecated
- Index
- Help

*CT brush applet*

- Prev
- Next

- Frames
- No Frames

- All Classes

## Uses of Class org.alvaregordon.ctbrush.Main

- - ### Uses of Main in org.alvaregordon.ctbrush

    Constructors in org.alvaregordon.ctbrush with parameters of type Main

    | Constructor and Description |
    |  |
    | --- |
    | `MouseHandler(Main canvas)` This method creates a new mouse handler object for the CT brush canvas. |
    | `Workspace(Main canvas, int width, int height, int[] hidden)` Creates a new workspace from a 2D hidden image (represented as a 1D array object; indices are computed as [Y \* width + X]). |

- Package
- Class
- Use
- Tree
- Deprecated
- Index
- Help

*CT brush applet*

- Prev
- Next

- Frames
- No Frames

- All Classes

*Copyright © 2012 University of Manitoba.*
