# Supplementary material for: CT brush and CancerZap!: two video games for computed tomography dose minimization
Source: Theor Biol Med Model. 2015 May 12;12:7. doi: 10.1186/s12976-015-0003-4 (PMC4469010; doi:10.1186/s12976-015-0003-4)
Supplement: Additional file 3: — The file ctdocs.zip is a zip file that contains all of the JavaDoc API documentation for the CT Brush project. All of the JavaDoc API documentation is in HTML format. To view this documentation, please load index.html (contained within this file) into a web-browser. [file 12976_2015_3_MOESM3_ESM.zip › docs/org/alvaregordon/ctbrush/class-use/GFXMath.html]

Uses of Class org.alvaregordon.ctbrush.GFXMath


JavaScript is disabled on your browser.


- Package
- Class
- Use
- Tree
- Deprecated
- Index
- Help

*CT brush applet*

- Prev
- Next

- Frames
- No Frames

- All Classes

## Uses of Class org.alvaregordon.ctbrush.GFXMath

No usage of org.alvaregordon.ctbrush.GFXMath

- Package
- Class
- Use
- Tree
- Deprecated
- Index
- Help

*CT brush applet*

- Prev
- Next

- Frames
- No Frames

- All Classes

*Copyright © 2012 University of Manitoba.*
