# Supplementary material for: CT brush and CancerZap!: two video games for computed tomography dose minimization
Source: Theor Biol Med Model. 2015 May 12;12:7. doi: 10.1186/s12976-015-0003-4 (PMC4469010; doi:10.1186/s12976-015-0003-4)
Supplement: Additional file 3: — The file ctdocs.zip is a zip file that contains all of the JavaDoc API documentation for the CT Brush project. All of the JavaDoc API documentation is in HTML format. To view this documentation, please load index.html (contained within this file) into a web-browser. [file 12976_2015_3_MOESM3_ESM.zip › docs/serialized-form.html]

Serialized Form


JavaScript is disabled on your browser.


- Package
- Class
- Use
- Tree
- Deprecated
- Index
- Help

*CT brush applet*

- Prev
- Next

- Frames
- No Frames

- All Classes

# Serialized Form

- ## Package org.alvaregordon.ctbrush

  - ### Class org.alvaregordon.ctbrush.Main extends javax.swing.JApplet implements Serializable

    - ### Serialized Fields

      - #### level

        ```
        int level
        ```

        The current level. Each level defines both the dimension for the canvas,
        and the maximum shape density for each canvas.
      - #### gcircles

        ```
        int gcircles
        ```

        The correct number of gray circles in the picture (if the user guesses or
        determines this number, they win!)
      - #### gfxout

        ```
        java.awt.Graphics gfxout
        ```

        The graphics object for 'canvas'.
      - #### work

        ```
        Workspace work
        ```

        The data workspace, which houses all of the CT brush image arrays.
      - #### canvasImage

        ```
        java.awt.image.BufferedImage canvasImage
        ```

        The canvas displayed to the user.
        The pixels in the canvas should be mostly the same as the pixel
        array 'work'. The exception being that the canvas also contains
        the mouse cursor. The reason for this separation is that Java does
        not easily allow lines to be erased.
        The canvas can also be used to show the hidden image to the user.
      - #### BRUSH\_BAR

        ```
        javax.swing.JToolBar BRUSH_BAR
        ```

        The toolbar to display the CT program and brush options.
      - #### BRUSH\_ICON

        ```
        javax.swing.ImageIcon BRUSH_ICON
        ```

        The image icon object to display the canvas to the user.
      - #### BRUSH\_AREA

        ```
        javax.swing.JLabel BRUSH_AREA
        ```

        The JLabel to display the canvas to the user.
      - #### SCROLL\_AREA

        ```
        javax.swing.JScrollPane SCROLL_AREA
        ```

        The JScrollPane to enable scrolling for the user.
      - #### SELF

        ```
        javax.swing.JApplet SELF
        ```

        A self-reference to the JApplet object. For use in inner-classes.
      - #### NEW\_ACTION

        ```
        javax.swing.AbstractAction NEW_ACTION
        ```

        This button starts a new game.
      - #### DONE\_ACTION

        ```
        javax.swing.AbstractAction DONE_ACTION
        ```

        The Finish image button. This button finishes the current game by asking
        the user a challenge question, determining if the user has won, and then
        counting the final score.
      - #### HELP\_ACTION

        ```
        javax.swing.AbstractAction HELP_ACTION
        ```

        This button displays the help dialog box to the user.
      - #### REFINE\_ACTION

        ```
        javax.swing.AbstractAction REFINE_ACTION
        ```

        A menu item for performing refinement iterations on the canvas data.
      - #### MOUSE\_HANDLER

        ```
        MouseHandler MOUSE_HANDLER
        ```

        The mouse handler. This object handles all mouse events for the program.
      - #### STATUS\_BAR

        ```
        javax.swing.JLabel STATUS_BAR
        ```

        The status bar for the CT-brush program
      - #### sockout

        ```
        java.io.PrintWriter sockout
        ```

        The server resource to write user movements to. This is a socket on the
        server where the user's mouse clicks will be sent. These clicks may be
        analyzed later to determine how the user deduced the final image, and use
        their strategies to help improve CT scan algorithms. The track feature
        may be disabled by not setting the Applet "track" parameter, or setting
        it to whitespace.

- Package
- Class
- Use
- Tree
- Deprecated
- Index
- Help

*CT brush applet*

- Prev
- Next

- Frames
- No Frames

- All Classes

*Copyright © 2012 University of Manitoba.*
