# Supplementary material for: CT brush and CancerZap!: two video games for computed tomography dose minimization
Source: Theor Biol Med Model. 2015 May 12;12:7. doi: 10.1186/s12976-015-0003-4 (PMC4469010; doi:10.1186/s12976-015-0003-4)
Supplement: Additional file 3: — The file ctdocs.zip is a zip file that contains all of the JavaDoc API documentation for the CT Brush project. All of the JavaDoc API documentation is in HTML format. To view this documentation, please load index.html (contained within this file) into a web-browser. [file 12976_2015_3_MOESM3_ESM.zip › docs/index-all.html]

Index


JavaScript is disabled on your browser.


- Package
- Class
- Use
- Tree
- Deprecated
- Index
- Help

*CT brush applet*

- Prev
- Next

- Frames
- No Frames

- All Classes

A B C D F G H I M N O R S U W 


## A

addProjection(int, int) - Method in class org.alvaregordon.ctbrush.Workspace
:   Adds a projection to the workspace.


## B

BRUSH\_AREA - Variable in class org.alvaregordon.ctbrush.Main
:   The JLabel to display the canvas to the user.


## C

circleBresenhamFloat(int[], int, int, int, int, int, boolean) - Static method in class org.alvaregordon.ctbrush.GFXMath
:   Bresenhanm Circle algorithm adapted from:
    http://www.sunshine2k.de/coding/java/Bresenham/RasterisingLinesCircles.pdf
    http://stackoverflow.com/questions/1201200/fast-algorithm-for-drawing-filled-circles
    http://en.wikipedia.org/wiki/Midpoint\_circle\_algorithm


## D

displayImage(BufferedImage) - Method in class org.alvaregordon.ctbrush.Workspace
:   Display the current workspace image in the user's canvas.

do\_projection(int, int, int, float[], int[]) - Static method in class org.alvaregordon.ctbrush.GFXMath
:   This function executes the MART algorithm using a given projection.

drawCursor(Graphics, int, int) - Method in class org.alvaregordon.ctbrush.MouseHandler
:   Draws the brush at the current cursor position, so the user can
    see what they are doing - they can see what area clicking the mouse
    should uncover.

drawLine(int[], int, int, int, int, int, int, boolean) - Static method in class org.alvaregordon.ctbrush.GFXMath
:   Digital Differential Analyzer line algorithm adapted from:
    http://www.sunshine2k.de/coding/java/Bresenham/RasterisingLinesCircles.pdf


## F

fillBottomFlatTriangle(int[], int, int, int, int, int, int, int, int, boolean) - Static method in class org.alvaregordon.ctbrush.GFXMath
:   Draws a triangle, which is flat at the bottom.

fillTopFlatTriangle(int[], int, int, int, int, int, int, int, int, boolean) - Static method in class org.alvaregordon.ctbrush.GFXMath
:   Draws a triangle, which is flat at the top.

finalize() - Method in class org.alvaregordon.ctbrush.Workspace


## G

getCanvasLocation() - Method in class org.alvaregordon.ctbrush.Main


getDose() - Method in class org.alvaregordon.ctbrush.Workspace
:   Calculates the total amount of dose administered for the current leve.

getNrays() - Method in class org.alvaregordon.ctbrush.MouseHandler


getWrays() - Method in class org.alvaregordon.ctbrush.MouseHandler


GFXMath - Class in org.alvaregordon.ctbrush
:   SYNOPSIS
    This class contains all of the mathematical algorithms for
    manipulating the hidden canvas (used by Main.genMap to generate
    the hidden canvas for each level), and the MART algorithm for
    brushing the workspace canvas.

GFXMath() - Constructor for class org.alvaregordon.ctbrush.GFXMath


## H

hasProjection(int, short) - Method in class org.alvaregordon.ctbrush.Main
:   Tests if a projection has already been "shot" (read) from the workspace.

hasProjection(int, int) - Method in class org.alvaregordon.ctbrush.Workspace
:   Checks if a projection was already added to the workspace.


## I

init() - Method in class org.alvaregordon.ctbrush.Main
:   Initializes the CT brush Applet.


## M

Main - Class in org.alvaregordon.ctbrush
:   SYNOPSIS

Main() - Constructor for class org.alvaregordon.ctbrush.Main


mouseBrush(int, int, byte, short, byte) - Method in class org.alvaregordon.ctbrush.Main
:   Performs a mouse CT-brush operation

mouseClicked(MouseEvent) - Method in class org.alvaregordon.ctbrush.MouseHandler
:   Performs a CT brush event when the mouse is clicked.

mouseDragged(MouseEvent) - Method in class org.alvaregordon.ctbrush.MouseHandler
:   Handles dragging the mouse.

MouseHandler - Class in org.alvaregordon.ctbrush
:   SYNOPSIS
    The mouse handler.

MouseHandler(Main) - Constructor for class org.alvaregordon.ctbrush.MouseHandler
:   This method creates a new mouse handler object for the CT brush canvas.

mouseMoved(MouseEvent) - Method in class org.alvaregordon.ctbrush.MouseHandler
:   Draws the brush at the current cursor position, so the user can see what
    they are doing - they can see what area clicking the mouse should
    uncover.

mouseReleased(MouseEvent) - Method in class org.alvaregordon.ctbrush.MouseHandler
:   Clean up interpolation for dragging.

mouseWheelMoved(MouseWheelEvent) - Method in class org.alvaregordon.ctbrush.MouseHandler
:   Sense mouse wheel movements and increment or decrement the number of rays
    in the mouse cursor.


## N

NRAY\_MAX - Static variable in class org.alvaregordon.ctbrush.MouseHandler
:   The maximum number of X-ray projections that can be used in the brush.

NRAY\_MIN - Static variable in class org.alvaregordon.ctbrush.MouseHandler
:   The minimum number of X-ray projections that can be used in the brush.

NRAY\_MINUS - Variable in class org.alvaregordon.ctbrush.MouseHandler
:   The increase number of rays button.

NRAY\_PLUS - Variable in class org.alvaregordon.ctbrush.MouseHandler
:   The increase number of rays button.

NRAY\_STEP - Static variable in class org.alvaregordon.ctbrush.MouseHandler
:   The increment step for number of X-ray projections to use in the brush.


## O

org.alvaregordon.ctbrush - package org.alvaregordon.ctbrush


## R

refine(Frame) - Method in class org.alvaregordon.ctbrush.Workspace
:   Performs a refinement MART iteration on the canvas.

REFINE\_ACTION - Variable in class org.alvaregordon.ctbrush.Main
:   A menu item for performing refinement iterations on the canvas data.

run() - Method in class org.alvaregordon.ctbrush.Workspace
:   Performs MART operations (based on the user's mouse clicks) in a
    separate thread.


## S

SELF - Variable in class org.alvaregordon.ctbrush.Main
:   A self-reference to the JApplet object.

showHidden(BufferedImage) - Method in class org.alvaregordon.ctbrush.Workspace
:   Display the hidden image for the workspace

SLOPES - Static variable in class org.alvaregordon.ctbrush.GFXMath
:   The tangent and co-tangents for every degree angles possible within
    the brush.


## U

updateImage(boolean) - Method in class org.alvaregordon.ctbrush.Main
:   Updates the current canvas image.

updateStatus() - Method in class org.alvaregordon.ctbrush.Main


## W

Workspace - Class in org.alvaregordon.ctbrush
:   SYNOPSIS
    The workspace canvas for the CT brush.

Workspace(Main, int, int, int[]) - Constructor for class org.alvaregordon.ctbrush.Workspace
:   Creates a new workspace from a 2D hidden image (represented as a 1D
    array object; indices are computed as [Y \* width + X]).

WRAY\_MAX - Static variable in class org.alvaregordon.ctbrush.MouseHandler
:   The maximum width of X-ray projections that can be used in the brush.

WRAY\_MIN - Static variable in class org.alvaregordon.ctbrush.MouseHandler
:   The minimum width of X-ray projections that can be used in the brush.

WRAY\_MINUS - Variable in class org.alvaregordon.ctbrush.MouseHandler
:   The decrease width of X-rays button.

WRAY\_PLUS - Variable in class org.alvaregordon.ctbrush.MouseHandler
:   The increase width of X-rays button.

WRAY\_STEP - Static variable in class org.alvaregordon.ctbrush.MouseHandler
:   The increment step for width of X-ray projections to use in the brush.

A B C D F G H I M N O R S U W

- Package
- Class
- Use
- Tree
- Deprecated
- Index
- Help

*CT brush applet*

- Prev
- Next

- Frames
- No Frames

- All Classes

*Copyright © 2012 University of Manitoba.*
