# Supplementary material for: CT brush and CancerZap!: two video games for computed tomography dose minimization
Source: Theor Biol Med Model. 2015 May 12;12:7. doi: 10.1186/s12976-015-0003-4 (PMC4469010; doi:10.1186/s12976-015-0003-4)
Supplement: Additional file 3: — The file ctdocs.zip is a zip file that contains all of the JavaDoc API documentation for the CT Brush project. All of the JavaDoc API documentation is in HTML format. To view this documentation, please load index.html (contained within this file) into a web-browser. [file 12976_2015_3_MOESM3_ESM.zip › docs/index.html]

Generated Documentation (Untitled)


<noscript>
<div>JavaScript is disabled on your browser.</div>
</noscript>
<h2>Frame Alert</h2>
<p>This document is designed to be viewed using the frames feature. If you see this message, you are using a non-frame-capable web client. Link to <a href="org/alvaregordon/ctbrush/package-summary.html">Non-frame version</a>.</p>
